# PTPRD mutation is a prognostic biomarker for sensitivity to ICIs treatment in advanced non-small cell lung cancer

**DOI:** 10.18632/aging.204964

**Published:** 2023-08-18

**Authors:** Zhixuan Ren, Li Wang, Chaohui Leng

**Affiliations:** 1 Department of Radiation Oncology, Huadong Hospital, Fudan University, Shanghai 200433, P.R. China; 2 Department of Medical Oncology, Shanghai Pulmonary Hospital and Thoracic Cancer Institute, Tongji University School of Medicine, Shanghai 200433, P.R. China; 3 Department of Oncology, Jiujiang University Affilliated Hospital, Jiujiang 332000, P.R. China

**Keywords:** NSCLC, PTPRD, immune checkpoint inhibitors, nomogram, TCGA

## Abstract

Background: Immune checkpoint inhibitors (ICIs) have become the standard treatment for advanced non-small cell lung cancer (NSCLC). ICIs can provide durable responses and prolong survival for some patients. With the increasing routine of next-generation sequencing (NGS) in clinical practice, it is essential to integrate prognostic factors to establish novel nomograms to improve clinical prediction ability in NSCLC with ICIs treatment.

Methods: Clinical information, response data, and genome data of advanced NSCLC treated ICIs were obtained from cBioPortal. The top 20 gene alterations in durable clinical benefit (DCB) were compared with those genes in no durable benefit (NDB). Survival analyses were performed using the Kaplan-Meier plot method and selected clinical variables to develop a novel nomogram.

Results: The mutation of PTPRD was significantly related to progression free survival (PFS) and overall survival (OS) in advanced NSCLC with ICIs treatment (PFS: p = 0.0441, OS: p = 0.0086). The PTPRD mutation was closely related to tumor mutational burden (TMB) and tumor-infiltrating immune cells (TIICs). Two novel nomograms were built to predict the PFS and OS of advanced NSCLC patients with ICIs treatment.

Conclusions: Our study suggested that PTPRD mutations could serve as a predictive biomarker for the sensitivity to ICIs treatment and PFS and OS in advanced NSCLC with ICIs. Our systematic nomograms showed great potential value in clinical application to predict the PFS and OS for advanced NSCLC patients with ICIs.

## INTRODUCTION

Among various cancers, Lung cancer was the most incident and lethal malignant tumor worldwide [1]. Lung cancer could be split into two categories: small cell lung cancer (SCLC, 15%) and NSCLC (85%) [2]. Although the efficacy of treatment for advanced NSCLC remains poor, the prognosis of NSCLC increased gradually during recent years because of the ICIs [3–6]. ICIs have shown convincing clinical benefits, significantly prolonging the survival of advanced NSCLC patients. However, the response rate of NSCLC with ICIs is only about 20%, indicating that most NSCLC patients failed to respond to ICIs [7]. In clinical application, it is the greatest challenge to be found potential biomarkers to predict the efficacy of ICIs treatment and find the appropriate population. Therefore, it is extremely important to estimate biomarkers to identify advanced NSCLC patients who may respond to ICIs.

At present, there are some biomarkers related to ICIs that have been approved by many clinical trials, including PD-L1 expression, TMB, and MSI-H [8, 9]. Most clinical trials have shown increased response rates and prognosis in NSCLC with higher PD-L1 expression, but enrichment of responses is incomplete [9, 10]. Only PD-L1 expression is insufficient to meet the accuracy requirements of clinical application in NSCLC. Moreover, there is still a lack of standardization and consistency in PD-L1 detection methods. Nowadays, TMB has been regarded as an independent predictive biomarker of response to ICIs in many cancers, including NSCLC [11–13]. For most cancers, high TMB is related to improved survival in patients with ICIs treatment, but there has not been an internationally accepted definition of high TMB at present [14]. Moreover, several potential biomarkers have been reported in numerous studies, including tumor-infiltrating lymphocytes, gut microbiota, tumor driver gene mutation, and so on [15, 16]. Besides that, some clinical indicators of advanced NSCLC patients can also be used to predict the prognosis of immunotherapy. ICIs have been significantly less successful in never-smokers, including EGFR-mutated, ALK-rearranged, and other rarer oncogenic drivers of NSCLC [17, 18]. With the increasing routine of NGS in clinical practice, it is essential to integrate various clinical indicators and genome data to develop a prognostic nomogram for predicting the clinical outcomes (including PFS and OS) in patients receiving ICIs.

In this study, genomic and clinical data of advanced NSCLC treated with ICIs were downloaded from cBioPortal [12, 13, 19, 20]. Subsequently, we compared the top 20 gene alterations between the DCB and NDB groups. The correlation between the gene mutation and TIICs and TMB was investigated by TCGA cohort. Finally, two novel nomograms based on the clinicopathological features and mutational data were built to predict the PFS and OS in advanced NSCLC with ICIs.

## MATERIALS AND METHODS

### Data download

Three ICIs cohorts of advanced NSCLC with immunotherapy were obtained from cBioPortal. The first cohort (MSK, J Clin Oncol 2018) consisted of targeted NGS of 240 advanced NSCLC with anti-PD-(L)1 monotherapy or in combination with anti–CTLA-4 [19]. The second cohort (MSK, Science 2015) collected genomic and survival data of 16 advanced NSCLC with anti-PD-1 monotherapy [12]. The third cohort (MSKCC, Nat Genet 2019) consisted of 350 advanced NSCLC patients with anti-PD-(L)1 as monotherapy or in combination [13]. The efficacy of immunotherapy was assessed by RECIST version 1.1. Moreover, DCB was defined as CR/PR/SD that lasted > 6 months, and NDB was defined as PD or SD that lasted ≤ 6 months [21]. PFS was defined as the time from the initiation of immunotherapy to disease progression or death. OS was defined as the time from the initiation of immunotherapy to date of death or last follow-up. PD-L1 negative, weak, and strong were defined as the PD-L1 expression < 0, 50 > PD-L1 expression ≥ 1, and PD-L1 expression ≥ 50, respectively. The first and second ICIs cohorts contained PFS of advanced NSCLC, and the third cohort contained OS data. All clinical studies reported the assessment of TMB. Besides that, we conducted a comprehensive analysis of gene mutations and survival data of LUAD and LUSC cohorts from the TCGA database. cBioPortal data were shown in Supplementary Table 1.

### Construction of nomogram

Survival analyses were conducted using the Kaplan-Meier plot method and selected clinical variables to develop a novel nomogram. Based on the patient’s clinical data and other information, nomograms can predict the likelihood of an event, including PFS, OS, and recurrence. In this work, the construction of the nomogram was based on clinicopathological information and mutational data. The “rms” package, which stands for Regression Modeling Strategies, is a collection of functions that facilitates and simplifies regression modeling, testing, estimation, validation, plotting, prediction, and presentation by incorporating enhanced model design attributes. The “rms” package serves as a valuable tool for assisting and streamlining the modeling process. And the R package *rms* was used to construct the nomogram. And the predictive accuracy and discriminative value of nomogram mainly included the concordance index (C-index), calibration curve, and ROC curve.

### Bioinformatic analysis

GO is a common technique for studying the biological function of genetic data, which mainly includes biological process, cellular component, and molecular function [22]. KEGG database collects genomic, chemical, and systematic functional information [23]. In this study, GO and KEGG enrichment analyses were performed to top 20 mutation gene functional annotations using the R package clusterProfiler.

The gene mutation, gene expression, and clinical data of LUAD and LUSC were obtained from TCGA database. The TMB score was further calculated for each sample. According to the comparison between single gene mutation group and wild group, the different degree of whole genes was obtained, which was performed by the gene set enrichment analysis (GSEA). In addition, the relative abundance of TIICs in NSCLC was determined by converting the genes expression into the fraction of immune cells.

### Verification of the prognostic significance of gene

External validation of the prognostic significance of gene was performed by another ICIs cohort (MSK, Cancer Cell 2018). This cohort consisted of 75 NSCLC with anti-PD-1 combination with anti–CTLA-4 [20].

### Statistical analyses

In this work, GraphPad Prism 9.0 and R 4.2.2 were used for statistical analyses. One-way Student’s t tests were used to determine statistical significance. Survival analyses were performed using the Kaplan-Meier plot method and compared using the log-rank test. And *P*-value< 0.05 indicating statistically significant.

### Availability of data and materials

All data included in this study are available including cBioPortal of Cancer Genomics ([MSK, J Clin Oncol 2018], [MSK, Science 2015], [MSKCC, Nat Genet 2019], [MSK, Cancer Cell 2018]) and TCGA database.

## RESULTS

### Gene alterations associated with DCB

**Figure 1:**
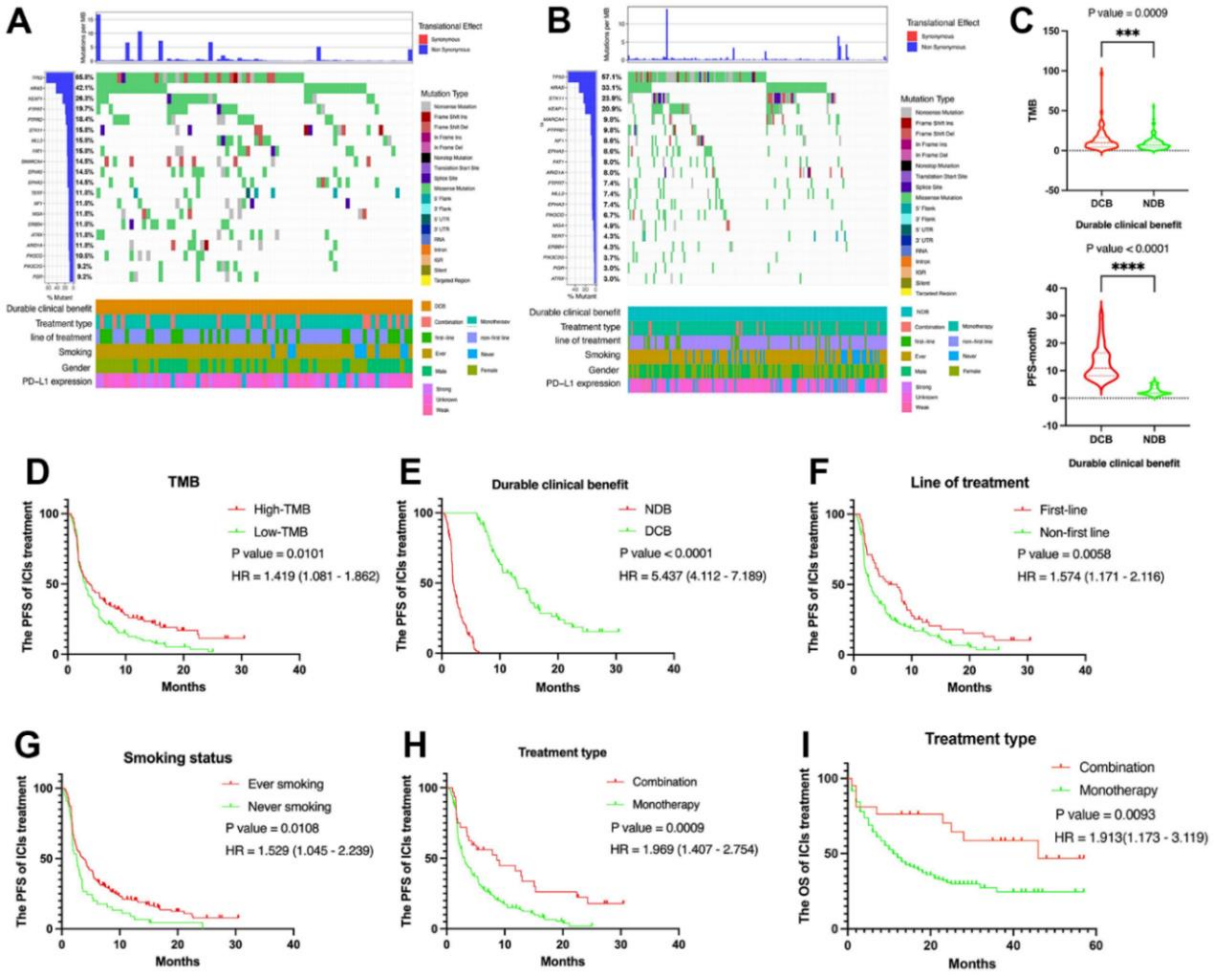
**Summary of genomic landscape and clinical features associated with response or non-response in NSCLC with immunotherapy.** (**A**, **B**) OncoPrint that has top 20 gene alterations in DCB group and NDB group. (**C**) TMB level and PFS were higher in DCB group than NDB group. (**D**) The Kaplan-Meier plot for NSCLC PFS based on TMB. (**E**) The Kaplan-Meier plot for NSCLC PFS based on PD-L1 expression. (**F**) The Kaplan-Meier plot for NSCLC PFS based on treatment line. (**G**) The Kaplan-Meier plot for NSCLC PFS based on smoking status. (**H**) The Kaplan-Meier plot for NSCLC PFS based on treatment type. (**I**) The Kaplan-Meier plot for NSCLC OS based on treatment type.

The top 20 gene alterations in DCB group include TP53, KRAS, KEAP1, PTPRT, PTPRD, STK11, MLL3, FAT1, SMARCA4, EPHA5, EPHA3, TERT, NF1, MGA, ERBB4, ATRX, ARID1A, PIK3CG, PIK3C2G, PGR (Figure 1A). We also examined those gene alterations in NDB group (Figure 1B). TMB (P = 0.0009) and PFS (P < 0.0001) were higher in DCB than NDB (Figure 1C). In addition, there are some clinicopathological variables were closely related to the PFS of ICIs including TMB (P = 0.0101, HR = 1.419 (1.081 – 1.862)), PD-L1 expression (P = 0.0062, HR = 1.799 (1.139 – 2.842)), line of treatment (P = 0.0058, HR = 1.574 (1.171 – 2.116)), smoking status (P = 0.0102, HR = 1.529 (1.045 –2.239)), and treatment type (P = 0.0009, HR = 1.969 (1.407 – 2.754)) (Figure 1D–1H). Moreover, treatment type (P value = 0.0093, HR = 1.913 (1.173 – 3.119)) was significantly related to the OS in advanced NSCLC patients with ICIs treatment (Figure 1I). And patient characteristics with NDB and DCB groups were shown in Table 1.

**Table 1:** NSCLC patient characteristics of cBioPortal with ICIs in DCB and NDB group.

**Clinical characteristics**	**NDB group**	**DCB group**
**Total cases**	167	76
**Gender**		
Female	82 (49.1%)	42 (55.2%)
Male	85 (50.9%)	34 (44.7%)
**Age**		
<65	77 (46.1%)	44 (57.9%)
>=65	90 (53.9%)	32 (42.1%)
**Smoking**		
Ever	131 (78.4%)	67 (88.2%)
Never	36 (21.6%)	9 (11.8%)
**Treatment type**		
Monotherapy	151 (90.4%)	53 (69.7%)
Combination	16 (9.6%)	16 (21.1%)
**Line of treatment**		
First-line	25 (15.0%)	27 (35.5%)
Non-first line	142 (85.0%)	49 (64.5%)
**PD-L1 expression**		
Negative	35 (20.9%)	9 (11.8%)
Weak	19 (11.4%)	9 (11.8%)
Strong	8 (4.8%)	13 (17.1%)
Unknown	105 (62.9%)	45 (59.3%)
**TMB**		
High	74 (44.3%)	49 (64.5%)
Low	93 (55.7%)	27 (35.5%)

### Enrichment analysis

**Figure 2:**
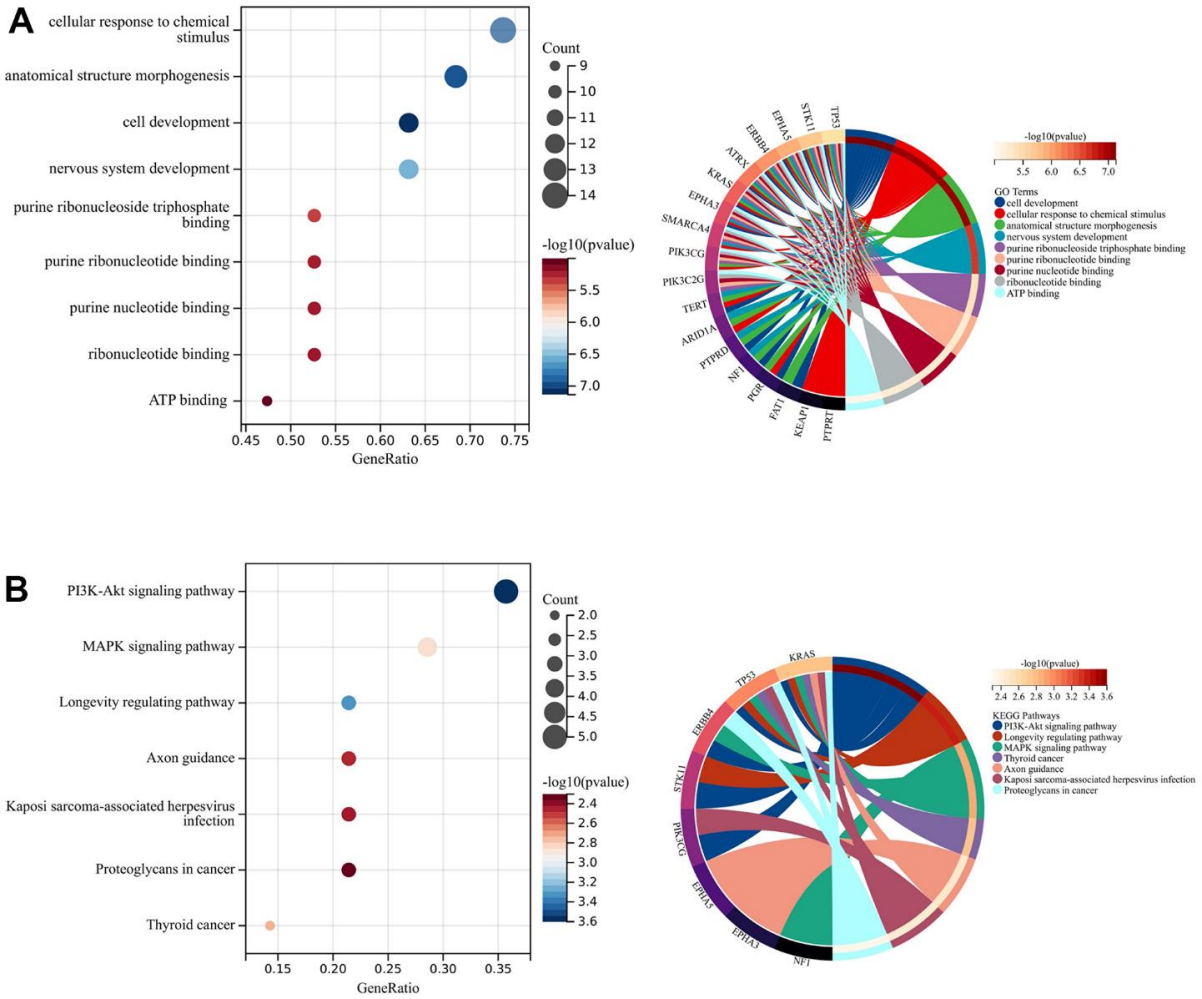
**Enrichment analysis and PPI network construction.** (**A**) GO analysis. (**B**) KEGG analysis.

The results of GO analysis suggested that the significant enrichment of the top 20 genes was mainly associated with cellular response to chemical stimulus, anatomical structure morphogenesis, cell development, and nervous system development (Figure 2A). In addition, three pathways were particularly enriched including PI3K-Akt signaling pathway, MAPK signaling pathway, and Longevity regulating pathway (Figure 2B). These pathways are all associated with tumor development and progression.

### Identification of survival related gene

**Figure 3:**
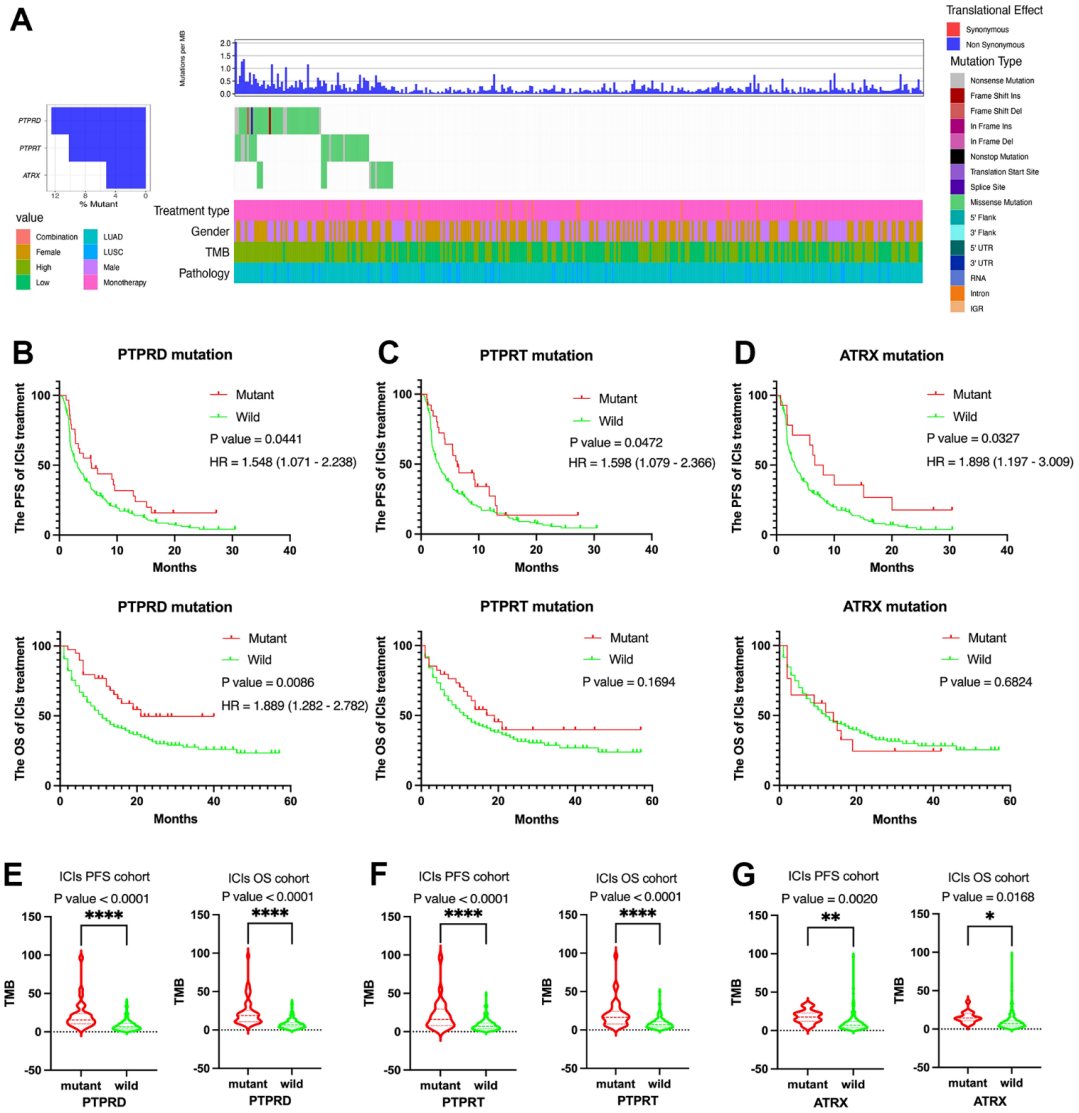
**Clinical and molecular features of ATRX, PTPRD, and PTPRT in advanced NSCLC with ICIs treatment.** (**A**) The prevalence of ATRX, PTPRD, and PTPRT mutations in the third cohort (MSKCC, Nat Genet 2019). (**B**) PTPRD was related to the PFS and OS. (**C**) PTPRT was related to the PFS. (**D**) ATRX was related to the PFS. (**E**–**G**) ATRX, PTPRD, and PTPRT mutations were closely related to higher TMB value.

Based on gene mutation and survival data, we selected the survival related gene from top 20 alterations in DCB group by the Kaplan-Meier plot method. ATRX, PTPRD, and PTPRT were closely related to PFS of NSCLC with immunotherapy in the first cohort (MSK, J Clin Oncol 2018) and the second cohort (MSK, Science 2015). Next, we analyzed the mutation status of ATRX, PTPRD, and PTPRT in the third cohort (MSKCC, Nat Genet 2019) (Figure 3A). Mutation frequencies of ATRX, PTPRD, and PTPRT were 5.4%, 13.4%, and 11.4%, respectively. Only PTPRD mutation was significantly related to the PFS and OS in advanced NSCLC with immunotherapy (Figure 3B). There was no significant difference in OS between ATRX and PTPRT mutations (Figure 3C, 3D). In addition, there was no significant difference in survival between ATRX, PTPRD, and PTPRT mutations in TCGA cohort (Supplementary Figure 1A–1C). The mutation status of ATRX, PTPRD, and PTPRT were both closely associated with higher TMB values (Figure 3E–3G). Moreover, the ATRX, PTPRD, and PTPRT mutation was related to higher TMB value in TCGA (Supplementary Figure 1D–1F).

### Development of nomogram for the prognosis of immunotherapy

**Figure 4:**
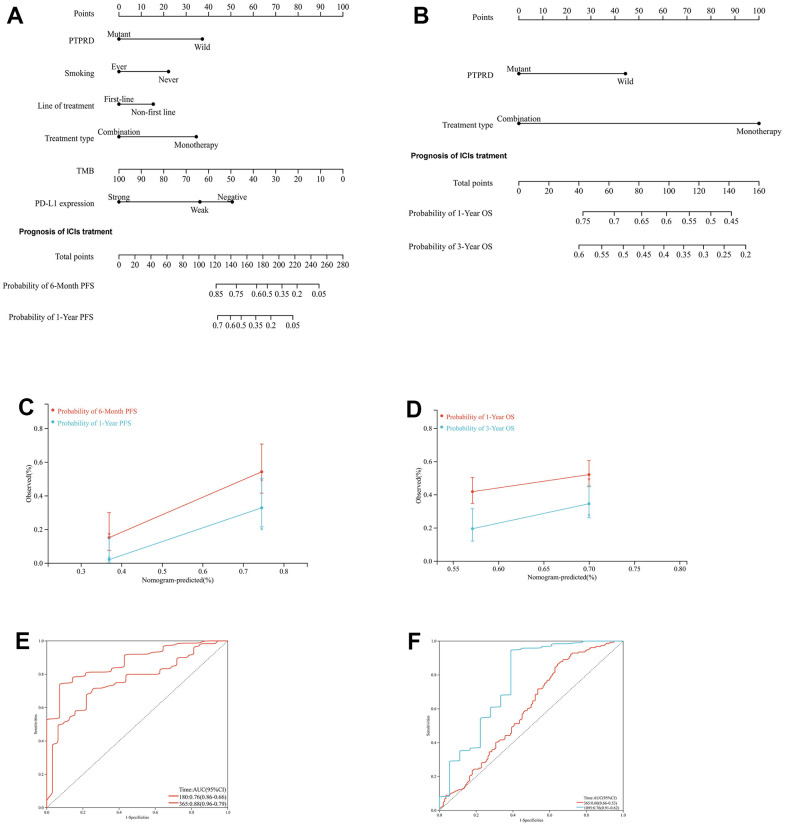
**Development of nomograms for the PFS and OS of NSCLC with ICIs.** (**A**) Systematic nomogram to predict the 6-month and 1-year PFS. (**B**) Systematic nomogram to predict the 1-year and 3-year OS. (**C**) The calibration plot for the chance of surviving 6-month and 1-year PFS. (**D**) The calibration plot for the chance of surviving 1-year and 3-year OS. (**E**) The ROC curve for the chance of 6-month and 1-year PFS. (**F**) The ROC curve for the chance of 6-month and 1-year PFS.

Survival analyses were performed using the Kaplan-Meier plot method and selected clinical variables to develop a novel nomogram. TMB (P value = 0.00101), PD-L1 expression (P value = 0.0062), line of treatment (P value = 0.0058), smoking status (P value = 0.00108), treatment type (P value = 0.0009) and PTPRD (P value = 0.0441) were significantly related to the PFS in advanced NSCLC patients with ICIs treatment (Figures 1D–1H, 3B). Based on these variables, we established a systematic nomogram to predict 6-month, and 1-year PFS for advanced NSCLC with ICIs (Figure 4A). The C-index of this nomogram was 0.680 (95% CI 0.617 to 0.742). The same, treatment type (P value = 0.0093) and PTPRD (P value = 0.0086) were significantly related to the OS in advanced NSCLC patients with ICIs treatment (Figures 1I, 3B). The survival probabilities for NSCLC patients with ICIs treatment at 1-year, and 3-year OS were predicted using a nomogram that was constructed based on these variables (Figure 4B). The C-index of this nomogram was 0.658 (95% CI 0.555 to 0.762). The calibration plot demonstrated great predictive performance of our two nomograms (Figure 4C, 4D). In addition, the ROC curve also showed a good ability of our two nomograms to predict 6-month PFS (AUC = 0.76), 1-year PFS (AUC =0.88), 1-year OS (AUC = 0.60), and 3-year OS (AUC = 0.76) of advanced NSCLC patients with immunotherapy (Figure 4E, 4F). Based on our nomograms, clinical physicians could obtain the total point based on all variable points, and then evaluate PFS and OS of each advanced NSCLC with immunotherapy.

### The role of PTPRD mutation in TIICs and GSEA

**Figure 5:**
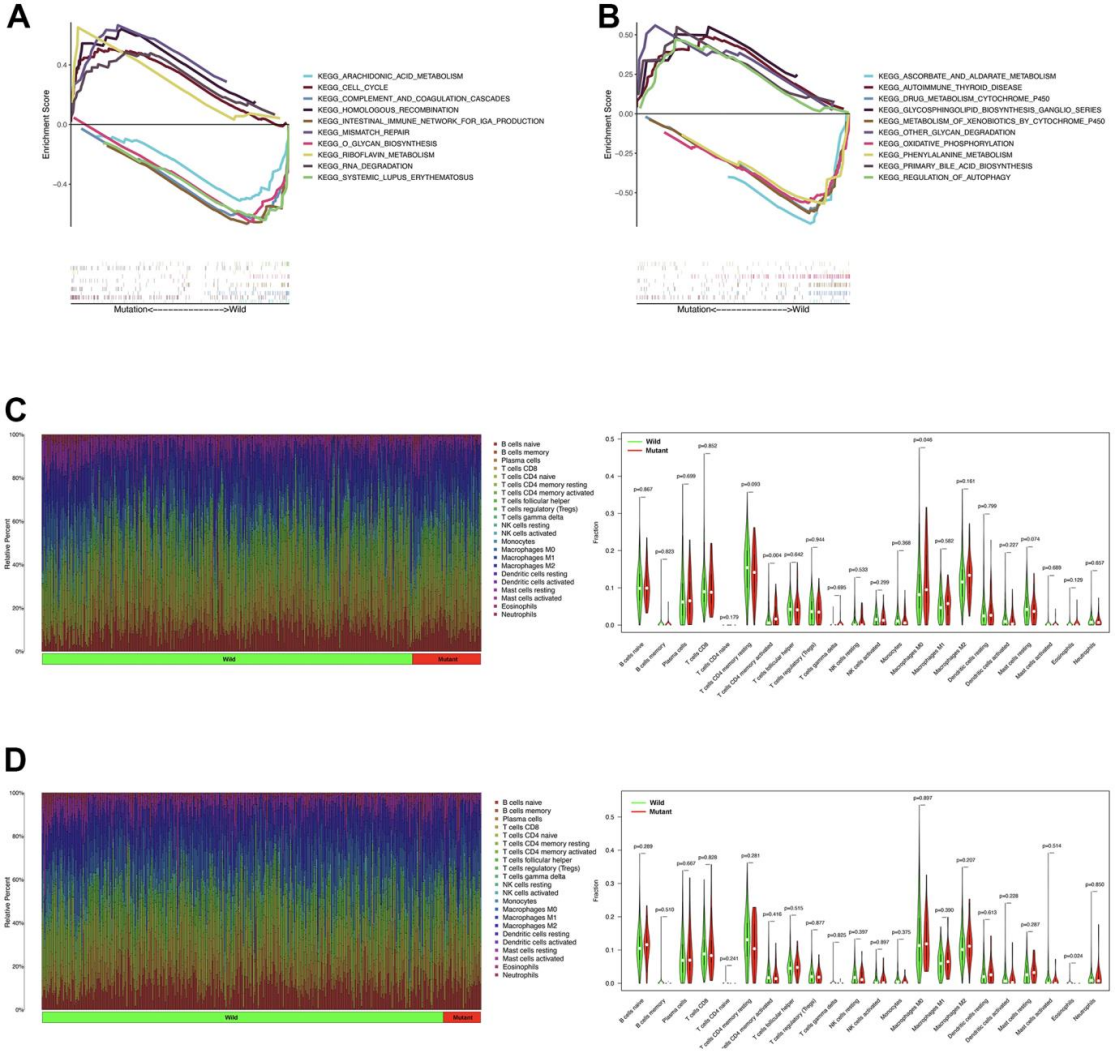
**The role of PTPRD mutation in GSEA and TIICs.** (**A**) GSEA analysis of PTPRD mutation in LUAD. (**B**) GSEA analysis of PTPRD mutation in LUSC. (**C**) The relationship between the mutation status of PTPRD and immune cells in LUAD. (**D**) The relationship between the mutation status of PTPRD and immune cells in LUSC.

The GSEA enrichment analysis performed with TCGA-LUAD cohort showed that riboflavin metabolism, RNA degradation, cell cycle, mismatch repair, and homologous recombination were significantly enriched in PTPRD mutation samples (Figure 5A). In TCGA-LUSC cohort, autoimmune thyroid disease, glycosphingolipid biosynthesis ganglio series, other glycan degradation, bile acid biosynthesis, and regulation of autophagy were significantly abundant in PTPRD mutation samples (Figure 5B).

We also evaluated the relationship between PTPRD mutation and TIICs in NSCLC. In TCGA-LUAD cohort, activated memory CD4 T cells, and M0 macrophages were more enriched in PTPRD mutant sample (Figure 5C). As for LUSC group, eosinophils were more abundant in PTPRD mutation sample (Figure 5D). The mutation of PTPRD may change the tumor microenvironment (TME), leading to the change of sensitivity to immunotherapy in NSCLC.

### Verification of the prognostic value of PTPRD mutation in immunotherapy

**Figure 6:**
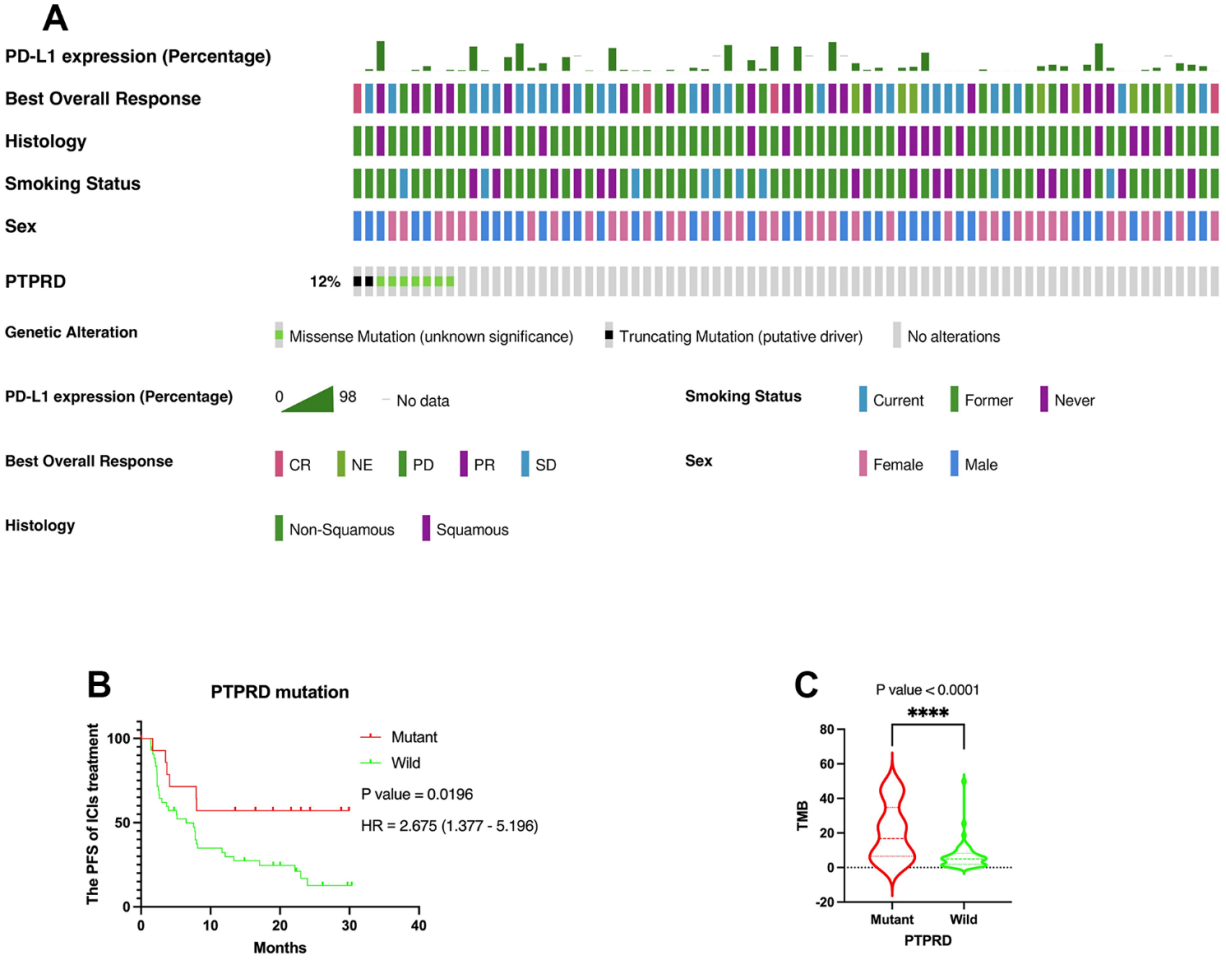
**Verification of the prognostic value of PTPRD in immunotherapy.** (**A**) Genomic landscape and clinical feature of PTPRD mutations in another ICIs cohort (MSK, Cancer Cell 2018). (**B**) The mutation of PTPRD was closely related to prognosis of LUAD with ICIs treatment. (**C**) PTPRD mutation was closely related to higher TMB in NSCLC.

We further explored the prognostic value of PTPRD mutation in another ICIs cohort (MSK, Cancer Cell 2018) as an independent external validation. Similarly, mutation frequency of PTPRD was 12% (Figure 6A). The same, PTPRD mutation was significantly related to the prognosis of LUAD with ICIs (P = 0.0196, HR = 2.675 (1.377 – 5.196)) (Figure 6B). PTPRD mutation was closely related to higher TMB value in NSCLC with ICIs (Figure 6C). Mutation frequency of PTPRD was 18.4% in DCB group (Figure 1A). However, mutation frequency of PTPRD was only 9.8% in NDB group (Figure 1B). According to the research above, we reasonably deduced that the mutation PTPRD can be used as a potential biomarker for the sensitivity to ICIs and prognosis of advanced NSCLC.

## DISCUSSION

At present, PD-L1 expression or TMB alone did not demonstrate a satisfactory ability to select advanced NSCLC patients who are likely to respond to ICIs. Apart from the expression of PD-L1 and TMB, there are some specific gene mutations including KRAS/TP53, STK11, EGFR, EPHA, and NOTCH, that are related to efficacy of ICIs by regulating the tumor microenvironment and served as potential biomarkers to predict the clinical benefits of immunotherapy [24–27]. Sun et al. found that ARID1A mutation could serve as predictive biomarkers for the prognosis of ICIs [28]. Besides that, some studies show ERBB4 mutation and FGFR4 mutation could serve as a potential biomarker for the prognosis of NSCLC with ICIs treatment [29, 30]. With the increasing routine of NGS in clinical practice, it is essential to explore predictors factors of response to immunotherapy. At the same time, it is important to integrate these prognostic factors to establish a novel systematic nomogram to improve clinical prediction ability in NSCLC with ICIs treatment.

PTPRD is a member of protein tyrosine phosphatases, which negatively regulate tyrosine phosphorylation [31]. It has been reported that PTPRD has a tumor suppressor function, and mutation of PTPRD may promote tumor growth [32]. A high frequency of gene deletions of PTPRD occurs in a variety of cancers [33, 34]. And other mechanisms that may contribute to PTPRD inactivation include point mutations and promoter region hypermethylation [35, 36]. A study found that PTPRD inactivation promotes tumor metastasis by induced CXCL8 in gastric cancer [37]. Recently, a study analyzed the data of 1745 NSCLC and elucidated the landscape of interaction effects among common co-mutations on the efficacy of ICIs [38]. Particularly, KRAS mutation remarkably interacted with its co-occurring mutations in TP53, STK11, PTPRD, RBM10, and ATM in non-squamous NSCLC. In addition, Shang et al. found that PTPRD and PTPRT mutations may be a biomarker for predicting immunotherapy in pan-cancer, but the role of PTPRD mutation in NSCLC has not been analyzed separately [39]. Li et al. suggested that PTPRD/PTPRT mutation was significantly associated with better progression-free survival (PFS) in three independent cohorts. The median PFS for PTPRD/PTPRT mutant-type vs. wild-type NSCLC patients were not reached vs. 6.3 months (Rizvi2015, HR = 0.16; 95% CI, 0.02-1.17; P=0.03), 24.0 vs. 5.4 months (Hellmann2018, HR, 0.49; 95% CI, 0.26-0.94; P=0.03), 5.6 vs. 3.0 months (Rizvi2018, HR = 0.64; 95% CI, 0.44-0.92; P=0.01) and 6.8 vs. 3.5 months (Pooled cohort, HR, 0.54; 95% CI, 0.39-0.73; P<0.0001) respectively [40]. Sun et al. demonstrated that tissue or circulating tumor DNA PTPRD mutation is a prognostic biomarker predicting prognosis of anti-PD-(L)1 monotherapy in non-squamous NSCLC patients [41]. However, the above study failed to predict OS in validation cohort, and squamous NSCLC were not included in the study. There is still a lack of sufficient literature to support the clinical prognostic significance of ICIs treatment in NSCLC patients who harbor PTPRD mutations. The underlying mechanisms of PTPRD mutation that regulate immune-related pathways in NSCLC remain unknown. According to these studies, we further explored the significance of PTPRD mutations in predicting the clinical outcome (PFS and OS) of advanced NSCLC with immunotherapy.

In this study, mutation frequencies of PTPRD in DCB group, NDB group, the third cohort (MSKCC, Nat Genet 2019), and the last cohort (MSK, Cancer Cell 2018) were 18.4%, 9.8%, 13.4%, and 12%, respectively. The Kaplan–Meier survival curves showed the mutation of PTPRD was associated with the PFS (P = 0.0441, HR = 1.548 (1.071 – 2.238)) and OS (P = 0.0086, HR = 1.889 (1.282 – 2.782)) in NSCLC with ICIs treatment. The prognostic value of PTPRD mutation was validated in the last ICIs cohort as an independent external validation (P = 0.0196, HR = 2.675 (1.377 – 5.196)). Besides, the PTPRD mutation was closely associated with higher TMB values in all ICIs cohort and TCGA cohort (P < 0.0001). Therefore, we reasonably deduced that the mutation PTPRD can be used as a potential biomarker for the sensitivity to ICIs treatment and prognosis of advanced NSCLC.

To explain why PTPRD mutations were associated with favorable ICIs prognosis, we evaluated the role of PTPRD mutation in GSEA and immune infiltration. The GSEA enrichment analysis showed that PTPRD mutation samples were mainly associated with riboflavin metabolism, RNA degradation, cell cycle, and mismatch repair in LUAD, and glycosphingolipid biosynthesis ganglio series, other glycan degradation, bile acid biosynthesis, and regulation of autophagy in LUSC, respectively. Besides that, we found that activated memory CD4 T cells, M0 macrophages, and eosinophils were more enriched in PTPRD mutant sample. Some studies revealed that eosinophils were closely associated with clinical outcomes in patients with ICIs treatment [42–44]. The mutation of PTPRD may influence CD4^+^ T cell infiltration via its phosphatase activity, and promote the occurrence of “hot” TME with enhanced anti-tumor immunity in advanced NSCLC patients.

Moreover, NSCLC patient’s clinical variables and genomic data that we included were screened by the Kaplan-Meier plot method. TMB, PD-L1 expression, line of treatment, smoking status, treatment type, and PTPRD mutant were significantly related to the PFS in advanced NSCLC patients with ICIs treatment. Besides that, treatment type and PTPRD mutant were significantly related to the OS in advanced NSCLC patients with ICIs treatment. According to the above results, we constructed two novel systematic nomograms that are based on the patient’s clinicopathological parameters and genome data to predict the prognosis for advanced NSCLC with ICIs. Based on our novel nomograms, clinicians can obtain the total point based on all variable points for each patient and can then predict the 6-month PFS, 1-year PFS, 1-year OS, and 3-year OS of advanced NSCLC patients with immunotherapy. Our novel nomograms can be used to assess the PFS and OS of NSCLC patients ICIs and formulate the appropriate type of treatment and follow-up time before immunotherapy.

The existing literature suggested that the mechanism of PTPRD mutation in NSCLC with ICIs still needs to be further explored. This study is the first to use the large database to establish two systematic nomograms for predicting the PFS and OS of advanced NSCLC patients with ICIs treatment, which undoubtedly provides a new clinical strategy for advanced NSCLC patients. This work is the first to illustrate the role of PTPRD mutations as a potential biomarker for the sensitivity to ICIs treatment and prognosis of advanced NSCLC. PTPRD can be used as a potential biomarker in regulating the TME and associating with the PFS and OS in advanced NSCLC with immunotherapy. Nevertheless, there remains some limitations in our work. First, this work employed four ICIs cohorts from the cBioPortal database. Due to the utilization of different platforms and detection depths, four cohorts may exhibit potential heterogeneity. Second, clinical information, response data, and genome data of NSCLC with ICIs were downloaded from four public datasets, some specific patients’ information and clinicopathological variables were unclear or not available. Third, four ICIs cohorts used different treatment types, such as immunotherapy monotherapy or combination with anti-CTLA-4, resulting in a certain degree of research heterogeneity. In addition, the mechanism of PTPRD affecting TME and immunotherapy in NSCLC needs further experiments to explore.

## CONCLUSIONS

To conclude, our study demonstrated that PTPRD mutation could serve as a prognostic biomarker predicting the PFS and OS in advanced NSCLC treated ICIs. PTPRD mutation is strongly associated with high TMB levels and immune infiltration and enhanced anti-tumor microenvironment. Our systematic nomograms showed great potential value in clinical application to predict the PFS and OS in advanced NSCLC with immunotherapy.

## Supplementary Material

Supplementary Figure 1

Supplementary Table 1
