# Correction for: PTPRD mutation is a prognostic biomarker for sensitivity to ICIs treatment in advanced non-small cell lung cancer

**DOI:** 10.18632/aging.206186

**Published:** 2024-12-31

**Authors:** Zhixuan Ren, Li Wang, Chaohui Leng

**Affiliations:** 1 Department of Radiation Oncology, Huadong Hospital, Fudan University, Shanghai 200433, P.R. China; 2 Department of Medical Oncology, Shanghai Pulmonary Hospital and Thoracic Cancer Institute, Tongji University School of Medicine, Shanghai 200433, P.R. China; 3 Department of Oncology, Jiujiang University Affiliated Hospital, Jiujiang 332000, P.R. China

**Keywords:** NSCLC, PTPRD, immune checkpoint inhibitors, nomogram, TCGA

**This article has been corrected:** The authors found that name of the last/corresponding author has been mistakenly recorded as Chaohui Leng, and the correct name should be Zhaohui Leng. In addition, the order of the author’s affiliations was also corrected.

The corrected version of the **authors list and affiliations** are provided below:

Zhixuan Ren^1,2,*^, Li Wang^3,*^, Zhaohui Leng^1^

^1^Department of Oncology, Jiujiang University Affiliated Hospital, Jiujiang 332000, P.R. China

^2^Department of Radiation Oncology, Huadong Hospital, Fudan University, Shanghai 200433, P.R. China

^3^Department of Medical Oncology, Shanghai Pulmonary Hospital and Thoracic Cancer Institute, Tongji University School of Medicine, Shanghai 200433, P.R. China

^*^Equal contribution

**Correspondence to:** Zhaohui Leng; **email: **huposetang@i.smu.edu.cn

