# Supplementary material for: PTPRD mutation is a prognostic biomarker for sensitivity to ICIs treatment in advanced non-small cell lung cancer
Source: Aging (Albany NY). 2023 Aug 18;15(16):8204–19. doi: 10.18632/aging.204964 (PMC10497019; doi:10.18632/aging.204964)
Supplement: Supplementary Figure 1 [file aging-15-204964-s001.pdf]

SUPPLEMENTARY FIGURE

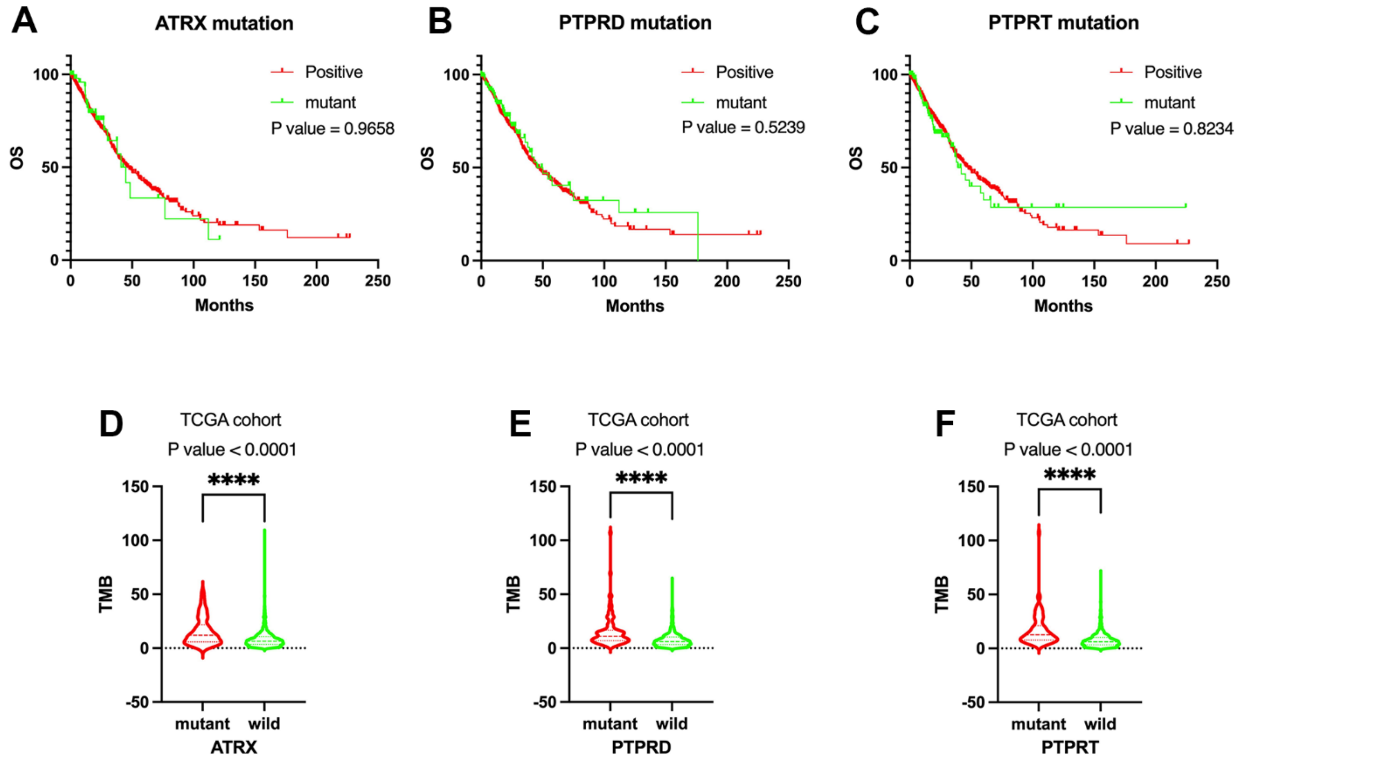

Supplementary Figure 1. Clinical feature of ATRX, PTPRD, and PTPRT mutations in TCGA cohort. (**A**–**C**) There was no significant difference of survival between ATRX, PTPRD, and PTPRT mutations. (**D**–**F**) ATRX, PTPRD, and PTPRT mutations were both closely associated with higher TMB value.
