# Supplementary material for: PTPRD mutation is a prognostic biomarker for sensitivity to ICIs treatment in advanced non-small cell lung cancer
Source: Aging (Albany NY). 2023 Aug 18;15(16):8204–19. doi: 10.18632/aging.204964 (PMC10497019; doi:10.18632/aging.204964)
Supplement: Supplementary Table 1 [file aging-15-204964-s002.docx]

**Supplementary Table 1. Patient characteristics of NSCLC with ICIs in DCB and NDB group.**

| PATIENT_ID | SAMPLE_ID | SAMPLE_COVERAGE | TUMOR_PURITY | ONCOTREE_CODE | SOMATIC_STATUS | AGE_AT_SEQ_REPORT | TMB_NONSYNONYMOUS | SEX | OS_MONTHS | OS_STATUS | AGE_GROUP | DRUG_TYPE |
| --- | --- | --- | --- | --- | --- | --- | --- | --- | --- | --- | --- | --- |
| P-0000082 | P-0000082-T01-IM3 | 905 | NA | LUAD | Matched | 61 | 13.30986 | Male | 57 | 0:LIVING | 50-60 | PD-1/PDL-1 |
| P-0000165 | P-0000165-T01-IM3 | 795 | 40 | LUAD | Unmatched | 67 | 5.545777 | Female | 1 | 1:DECEASED | 61-70 | PD-1/PDL-1 |
| P-0000205 | P-0000205-T01-IM3 | 1214 | 90 | LUAD | Unmatched | 56 | 5.545777 | Female | 2 | 1:DECEASED | 50-60 | Combo |
| P-0000208 | P-0000208-T01-IM3 | 1346 | 70 | LUAD | Matched | 69 | 2.218311 | Female | 13 | 1:DECEASED | 61-70 | PD-1/PDL-1 |
| P-0000235 | P-0000235-T01-IM3 | 618 | 70 | LUSC | Matched | 76 | 8.873242 | Male | 3 | 1:DECEASED | >71 | PD-1/PDL-1 |
| P-0000302 | P-0000302-T01-IM3 | 739 | 30 | LUSC | Matched | 65 | 1.109155 | Male | 15 | 0:LIVING | 61-70 | PD-1/PDL-1 |
| P-0000363 | P-0000363-T01-IM3 | 418 | 40 | LUSC | Matched | 71 | 12.20071 | Male | 2 | 1:DECEASED | >71 | PD-1/PDL-1 |
| P-0000458 | P-0000458-T01-IM3 | 1201 | 20 | LUAD | Matched | 66 | 1.109155 | Male | 5 | 1:DECEASED | 61-70 | PD-1/PDL-1 |
| P-0000563 | P-0000563-T01-IM3 | 369 | 45 | LUAD | Matched | 42 | 3.327466 | Male | 6 | 1:DECEASED | 31-50 | PD-1/PDL-1 |
| P-0000604 | P-0000604-T01-IM3 | 990 | 25 | LUAD | Matched | 59 | 4.436621 | Male | 57 | 0:LIVING | 50-60 | Combo |
| P-0000628 | P-0000628-T01-IM3 | 869 | 75 | LUAD | Matched | 52 | 4.436621 | Male | 2 | 1:DECEASED | 50-60 | PD-1/PDL-1 |
| P-0000670 | P-0000670-T01-IM3 | 438 | 35 | LUAD | Matched | 50 | 6.654932 | Female | 17 | 0:LIVING | 31-50 | Combo |
| P-0000681 | P-0000681-T01-IM3 | 834 | 75 | LUAD | Matched | 67 | 18.85564 | Female | 28 | 0:LIVING | 61-70 | PD-1/PDL-1 |
| P-0000702 | P-0000702-T01-IM3 | 605 | 50 | LUAD | Matched | 85 | 6.654932 | Female | 2 | 0:LIVING | >71 | PD-1/PDL-1 |
| P-0000731 | P-0000731-T01-IM3 | 549 | 80 | LUAD | Matched | 73 | 6.654932 | Female | 42 | 0:LIVING | >71 | PD-1/PDL-1 |
| P-0000867 | P-0000867-T01-IM3 | 364 | 50 | LUAD | Matched | 66 | 6.654932 | Male | 0 | 1:DECEASED | 61-70 | PD-1/PDL-1 |
| P-0000894 | P-0000894-T01-IM3 | 532 | 85 | LUAD | Matched | 80 | 5.545777 | Male | 57 | 0:LIVING | >71 | PD-1/PDL-1 |
| P-0000978 | P-0000978-T01-IM3 | 381 | 15 | LUAD | Matched | 58 | 1.109155 | Female | 7 | 1:DECEASED | 50-60 | PD-1/PDL-1 |
| P-0001093 | P-0001093-T01-IM3 | 540 | 15 | LUAD | Matched | 65 | 12.20071 | Male | 42 | 0:LIVING | 61-70 | Combo |
| P-0001121 | P-0001121-T01-IM3 | 728 | 90 | LUSC | Matched | 45 | 1.109155 | Female | 10 | 1:DECEASED | 31-50 | PD-1/PDL-1 |
| P-0001144 | P-0001144-T01-IM3 | 686 | 30 | LUSC | Matched | 64 | 7.764087 | Male | 2 | 1:DECEASED | 61-70 | PD-1/PDL-1 |
| P-0001171 | P-0001171-T01-IM3 | 1189 | 60 | LUAD | Matched | 52 | 96.49651 | Male | 29 | 0:LIVING | 50-60 | PD-1/PDL-1 |
| P-0001199 | P-0001199-T01-IM3 | 438 | 50 | LUAD | Matched | 76 | 14.41902 | Female | 2 | 1:DECEASED | >71 | PD-1/PDL-1 |
| P-0001296 | P-0001296-T01-IM3 | 303 | 40 | LUAD | Matched | 70 | 3.327466 | Female | 2 | 1:DECEASED | >71 | PD-1/PDL-1 |
| P-0001314 | P-0001314-T01-IM3 | 707 | 80 | LUAD | Matched | 44 | 2.218311 | Female | 1 | 1:DECEASED | 31-50 | PD-1/PDL-1 |
| P-0001365 | P-0001365-T01-IM3 | 683 | NA | LUAD | Matched | 70 | 5.545777 | Male | 2 | 1:DECEASED | >71 | PD-1/PDL-1 |
| P-0001385 | P-0001385-T01-IM3 | 580 | NA | LUAD | Matched | 51 | 14.41902 | Female | 40 | 0:LIVING | 31-50 | Combo |
| P-0001437 | P-0001437-T01-IM3 | 595 | 20 | LUAD | Matched | 65 | 1.109155 | Female | 4 | 1:DECEASED | 61-70 | PD-1/PDL-1 |
| P-0001633 | P-0001633-T01-IM3 | 758 | 40 | LUAD | Matched | 55 | 4.436621 | Female | 1 | 1:DECEASED | 50-60 | PD-1/PDL-1 |
| P-0001665 | P-0001665-T01-IM3 | 551 | 10 | LUAD | Matched | 48 | 1.109155 | Female | 5 | 1:DECEASED | 31-50 | PD-1/PDL-1 |
| P-0001832 | P-0001832-T01-IM3 | 534 | 30 | LUAD | Matched | 68 | 4.436621 | Female | 3 | 1:DECEASED | 61-70 | PD-1/PDL-1 |
| P-0001836 | P-0001836-T01-IM3 | 748 | 30 | LUAD | Matched | 60 | 25.51057 | Female | 6 | 0:LIVING | 50-60 | PD-1/PDL-1 |
| P-0001955 | P-0001955-T01-IM3 | 358 | 60 | LUAD | Matched | 31 | 2.218311 | Female | 6 | 1:DECEASED | 31-50 | PD-1/PDL-1 |
| P-0001977 | P-0001977-T01-IM3 | 159 | 40 | LUAD | Matched | 80 | 9.982398 | Male | 4 | 1:DECEASED | >71 | PD-1/PDL-1 |
| P-0002028 | P-0002028-T01-IM3 | 623 | default | LUSC | Matched | 65 | 6.654932 | Female | 33 | 0:LIVING | 61-70 | PD-1/PDL-1 |
| P-0002151 | P-0002151-T01-IM3 | 819 | 30 | LUAD | Matched | 71 | 9.982398 | Female | 25 | 0:LIVING | >71 | PD-1/PDL-1 |
| P-0002160 | P-0002160-T01-IM3 | 690 | 15 | LUAD | Matched | 67 | 13.30986 | Male | 6 | 1:DECEASED | 61-70 | PD-1/PDL-1 |
| P-0002225 | P-0002225-T01-IM3 | 1000 | 20 | LUSC | Matched | 72 | 1.109155 | Male | 17 | 1:DECEASED | >71 | PD-1/PDL-1 |
| P-0002247 | P-0002247-T01-IM3 | 628 | 30 | LUAD | Matched | 69 | 21.07395 | Female | 19 | 1:DECEASED | 61-70 | PD-1/PDL-1 |
| P-0002279 | P-0002279-T01-IM3 | 820 | 50 | LUSC | Matched | 70 | 7.764087 | Female | 3 | 1:DECEASED | 61-70 | PD-1/PDL-1 |
| P-0002295 | P-0002295-T01-IM3 | 892 | 30 | LUAD | Matched | 77 | 4.436621 | Female | 25 | 1:DECEASED | >71 | PD-1/PDL-1 |
| P-0002320 | P-0002320-T01-IM3 | 1494 | 20 | LUAD | Matched | 57 | 6.654932 | Male | 8 | 1:DECEASED | 50-60 | PD-1/PDL-1 |
| P-0002351 | P-0002351-T01-IM3 | 692 | 40 | LUSC | Matched | 73 | 7.764087 | Female | 11 | 1:DECEASED | >71 | PD-1/PDL-1 |
| P-0002444 | P-0002444-T01-IM3 | 737 | default | LUAD | Matched | 67 | 17.74648 | Male | 13 | 1:DECEASED | 61-70 | PD-1/PDL-1 |
| P-0002485 | P-0002485-T01-IM3 | 608 | default | LUSC | Matched | 45 | 3.327466 | Male | 24 | 1:DECEASED | 31-50 | PD-1/PDL-1 |
| P-0002546 | P-0002546-T01-IM3 | 282 | 20 | SARCL | Matched | 68 | 3.327466 | Male | 25 | 1:DECEASED | 61-70 | Combo |
| P-0002551 | P-0002551-T01-IM3 | 692 | default | LUAD | Matched | 67 | 17.74648 | Female | 6 | 0:LIVING | 61-70 | PD-1/PDL-1 |
| P-0002559 | P-0002559-T01-IM3 | 727 | default | LUAD | Matched | 39 | 1.109155 | Female | 43 | 0:LIVING | 31-50 | PD-1/PDL-1 |
| P-0002637 | P-0002637-T01-IM3 | 1086 | 50 | LUNE | Matched | 54 | 18.85564 | Male | 37 | 0:LIVING | 50-60 | Combo |
| P-0002639 | P-0002639-T01-IM3 | 760 | 30 | LUAD | Matched | 67 | 12.20071 | Female | 11 | 0:LIVING | 61-70 | PD-1/PDL-1 |
| P-0002646 | P-0002646-T01-IM3 | 885 | 20 | LUAD | Matched | 43 | 6.654932 | Male | 4 | 1:DECEASED | 31-50 | PD-1/PDL-1 |
| P-0002731 | P-0002731-T02-IM5 | 776 | 70 | LUAD | Matched | 64 | 9.787197 | Female | 25 | 0:LIVING | 61-70 | PD-1/PDL-1 |
| P-0002738 | P-0002738-T01-IM3 | 263 | 30 | LUNE | Matched | 32 | 7.764087 | Female | 1 | 1:DECEASED | 31-50 | PD-1/PDL-1 |
| P-0002751 | P-0002751-T01-IM3 | 91 | 20 | LUAD | Matched | 69 | 2.218311 | Female | 11 | 1:DECEASED | 61-70 | PD-1/PDL-1 |
| P-0002758 | P-0002758-T01-IM3 | 112 | 30 | LUSC | Matched | 63 | 35.49297 | Male | 2 | 1:DECEASED | 61-70 | PD-1/PDL-1 |
| P-0002766 | P-0002766-T01-IM3 | 757 | default | LUAD | Matched | 66 | 6.654932 | Female | 6 | 1:DECEASED | 61-70 | PD-1/PDL-1 |
| P-0002794 | P-0002794-T01-IM3 | 213 | 20 | LUAD | Unmatched | 61 | 0 | Female | 25 | 0:LIVING | 61-70 | PD-1/PDL-1 |
| P-0002806 | P-0002806-T01-IM3 | 613 | 20 | LUAD | Matched | 82 | 4.436621 | Male | 46 | 0:LIVING | >71 | PD-1/PDL-1 |
| P-0002826 | P-0002826-T01-IM3 | 777 | 40 | LUAD | Matched | 61 | 14.41902 | Female | 4 | 1:DECEASED | 61-70 | PD-1/PDL-1 |
| P-0002874 | P-0002874-T01-IM3 | 1045 | 40 | LUAD | Matched | 55 | 14.41902 | Female | 3 | 1:DECEASED | 50-60 | PD-1/PDL-1 |
| P-0002890 | P-0002890-T01-IM3 | 678 | 40 | LUAD | Matched | 40 | 6.654932 | Female | 0 | 1:DECEASED | 31-50 | PD-1/PDL-1 |
| P-0002905 | P-0002905-T01-IM3 | 430 | 20 | LUAD | Matched | 73 | 5.545777 | Male | 6 | 1:DECEASED | >71 | PD-1/PDL-1 |
| P-0002917 | P-0002917-T01-IM3 | 200 | 30 | LUAD | Matched | 51 | 6.654932 | Female | 30 | 0:LIVING | 50-60 | PD-1/PDL-1 |
| P-0002921 | P-0002921-T01-IM3 | 438 | 30 | LUAD | Matched | 73 | 14.41902 | Female | 12 | 1:DECEASED | >71 | PD-1/PDL-1 |
| P-0002965 | P-0002965-T01-IM3 | 970 | 20 | LUSC | Matched | 56 | 7.764087 | Male | 11 | 1:DECEASED | 50-60 | PD-1/PDL-1 |
| P-0002969 | P-0002969-T01-IM3 | 462 | 20 | LUAD | Matched | 56 | 22.18311 | Male | 2 | 1:DECEASED | 50-60 | PD-1/PDL-1 |
| P-0003034 | P-0003034-T01-IM5 | 1021 | default | LUAD | Matched | 80 | 13.70208 | Male | 5 | 1:DECEASED | >71 | PD-1/PDL-1 |
| P-0003119 | P-0003119-T01-IM5 | 951 | 30 | LUAD | Matched | 53 | 12.72336 | Male | 28 | 0:LIVING | 50-60 | PD-1/PDL-1 |
| P-0003132 | P-0003132-T01-IM5 | 620 | 30 | LUSC | Matched | 67 | 33.27647 | Female | 7 | 1:DECEASED | 61-70 | PD-1/PDL-1 |
| P-0003157 | P-0003157-T01-IM5 | 765 | 30 | LUSC | Matched | 63 | 5.872318 | Male | 3 | 1:DECEASED | 61-70 | PD-1/PDL-1 |
| P-0003229 | P-0003229-T01-IM5 | 550 | 60 | LUAD | Matched | 61 | 49.9147 | Male | 21 | 1:DECEASED | 50-60 | PD-1/PDL-1 |
| P-0003275 | P-0003275-T01-IM5 | 689 | 20 | LUAD | Matched | 69 | 2.936159 | Male | 17 | 1:DECEASED | 61-70 | PD-1/PDL-1 |
| P-0003278 | P-0003278-T01-IM5 | 589 | 50 | LUAD | Matched | 55 | 14.6808 | Female | 0 | 1:DECEASED | 50-60 | PD-1/PDL-1 |
| P-0003322 | P-0003322-T01-IM5 | 745 | 10 | LUAD | Matched | 72 | 2.936159 | Female | 13 | 0:LIVING | >71 | Combo |
| P-0003347 | P-0003347-T01-IM5 | 684 | 50 | LUSC | Matched | 59 | 15.65952 | Male | 15 | 1:DECEASED | 50-60 | PD-1/PDL-1 |
| P-0003368 | P-0003368-T01-IM5 | 743 | 20 | LUAD | Matched | 59 | 8.808477 | Female | 38 | 0:LIVING | 50-60 | Combo |
| P-0003495 | P-0003495-T01-IM5 | 530 | 30 | LUAD | Matched | 90 | 3.914879 | Female | 21 | 1:DECEASED | >71 | PD-1/PDL-1 |
| P-0003553 | P-0003553-T01-IM5 | 535 | 30 | LUAD | Matched | 70 | 29.36159 | Female | 21 | 0:LIVING | >71 | PD-1/PDL-1 |
| P-0003562 | P-0003562-T01-IM5 | 629 | 90 | LUAD | Matched | 62 | 36.21263 | Male | 45 | 0:LIVING | 50-60 | PD-1/PDL-1 |
| P-0003584 | P-0003584-T01-IM5 | 513 | 20 | LUAD | Matched | 61 | 10.76592 | Female | 36 | 1:DECEASED | 50-60 | PD-1/PDL-1 |
| P-0003628 | P-0003628-T01-IM5 | 788 | 40 | LUAD | Matched | 63 | 12.72336 | Male | 5 | 1:DECEASED | 61-70 | PD-1/PDL-1 |
| P-0003635 | P-0003635-T01-IM5 | 667 | 50 | LUAD | Matched | 57 | 6.851038 | Female | 2 | 1:DECEASED | 50-60 | Combo |
| P-0003670 | P-0003670-T01-IM5 | 662 | 30 | LUAD | Matched | 75 | 0.97872 | Female | 20 | 0:LIVING | >71 | PD-1/PDL-1 |
| P-0003738 | P-0003738-T02-IM5 | 859 | 70 | LUAD | Matched | 58 | 4.893598 | Female | 27 | 0:LIVING | 50-60 | PD-1/PDL-1 |
| P-0003782 | P-0003782-T01-IM5 | 708 | 10 | LUAD | Matched | 57 | 2.936159 | Male | 8 | 1:DECEASED | 50-60 | PD-1/PDL-1 |
| P-0003811 | P-0003811-T01-IM5 | 381 | 30 | LUSC | Matched | 68 | 16.63823 | Female | 0 | 1:DECEASED | 61-70 | PD-1/PDL-1 |
| P-0003869 | P-0003869-T01-IM5 | 643 | 40 | LUAD | Matched | 67 | 54.8083 | Female | 40 | 0:LIVING | 61-70 | PD-1/PDL-1 |
| P-0003913 | P-0003913-T01-IM3 | 818 | 30 | LUAD | Matched | 44 | 3.327466 | Female | 1 | 1:DECEASED | 31-50 | PD-1/PDL-1 |
| P-0003964 | P-0003964-T01-IM3 | 654 | 60 | LUAD | Matched | 72 | 9.982398 | Male | 4 | 1:DECEASED | >71 | PD-1/PDL-1 |
| P-0003967 | P-0003967-T01-IM5 | 481 | 20 | LUAD | Matched | 49 | 3.914879 | Female | 13 | 0:LIVING | 31-50 | PD-1/PDL-1 |
| P-0003970 | P-0003970-T01-IM5 | 481 | 40 | LUAD | Matched | 46 | 1.957439 | Female | 12 | 1:DECEASED | 31-50 | PD-1/PDL-1 |
| P-0003972 | P-0003972-T01-IM5 | 533 | 10 | SARCL | Matched | 65 | 5.872318 | Male | 0 | 1:DECEASED | 61-70 | PD-1/PDL-1 |
| P-0004020 | P-0004020-T01-IM5 | 693 | 20 | LUAD | Matched | 58 | 4.893598 | Female | 28 | 1:DECEASED | 50-60 | Combo |
| P-0004054 | P-0004054-T01-IM5 | 437 | 50 | LUAD | Matched | 61 | 13.70208 | Male | 4 | 1:DECEASED | 61-70 | PD-1/PDL-1 |
| P-0004099 | P-0004099-T01-IM5 | 652 | 40 | LUAD | Matched | 61 | 12.72336 | Male | 3 | 1:DECEASED | 61-70 | PD-1/PDL-1 |
| P-0004103 | P-0004103-T01-IM5 | 691 | 20 | LUAD | Matched | 44 | 0.97872 | Female | 23 | 1:DECEASED | 31-50 | Combo |
| P-0004117 | P-0004117-T01-IM5 | 734 | 40 | LUAD | Matched | 57 | 3.914879 | Male | 2 | 1:DECEASED | 50-60 | PD-1/PDL-1 |
| P-0004120 | P-0004120-T01-IM5 | 587 | 20 | LUAD | Matched | 58 | 0.97872 | Female | 12 | 0:LIVING | 50-60 | PD-1/PDL-1 |
| P-0004189 | P-0004189-T01-IM5 | 568 | 30 | LUAD | Matched | 71 | 58.72318 | Male | 16 | 0:LIVING | >71 | PD-1/PDL-1 |
| P-0004232 | P-0004232-T01-IM5 | 496 | 40 | LUAD | Matched | 75 | 6.851038 | Male | 18 | 1:DECEASED | >71 | PD-1/PDL-1 |
| P-0004279 | P-0004279-T01-IM5 | 544 | 40 | LUAD | Matched | 59 | 2.936159 | Female | 13 | 0:LIVING | 50-60 | PD-1/PDL-1 |
| P-0004315 | P-0004315-T01-IM5 | 686 | 20 | LUAD | Matched | 86 | 8.808477 | Female | 1 | 1:DECEASED | >71 | PD-1/PDL-1 |
| P-0004395 | P-0004395-T01-IM5 | 510 | 50 | LUAD | Matched | 65 | 7.829758 | Female | 0 | 1:DECEASED | 61-70 | PD-1/PDL-1 |
| P-0004436 | P-0004436-T01-IM5 | 836 | 40 | LUAD | Matched | 75 | 5.872318 | Male | 24 | 1:DECEASED | >71 | PD-1/PDL-1 |
| P-0004442 | P-0004442-T01-IM5 | 305 | 30 | LUAD | Matched | 62 | 34.25519 | Male | 26 | 0:LIVING | 61-70 | PD-1/PDL-1 |
| P-0004463 | P-0004463-T01-IM5 | 566 | 30 | LUAD | Matched | 83 | 6.851038 | Male | 13 | 1:DECEASED | >71 | PD-1/PDL-1 |
| P-0004472 | P-0004472-T01-IM5 | 201 | 30 | LUAD | Matched | 63 | 13.70208 | Female | 10 | 1:DECEASED | 61-70 | PD-1/PDL-1 |
| P-0004495 | P-0004495-T01-IM5 | 959 | 30 | LUSC | Matched | 40 | 8.808477 | Male | 8 | 1:DECEASED | 31-50 | PD-1/PDL-1 |
| P-0004517 | P-0004517-T01-IM5 | 726 | 50 | LUSC | Matched | 68 | 14.6808 | Male | 22 | 1:DECEASED | 61-70 | PD-1/PDL-1 |
| P-0004588 | P-0004588-T01-IM5 | 756 | 10 | LUAD | Matched | 73 | 3.914879 | Male | 30 | 0:LIVING | >71 | PD-1/PDL-1 |
| P-0004612 | P-0004612-T01-IM5 | 454 | 30 | LUAD | Matched | 74 | 3.914879 | Male | 4 | 1:DECEASED | >71 | PD-1/PDL-1 |
| P-0004613 | P-0004613-T01-IM5 | 1007 | 40 | LUAD | Matched | 61 | 3.914879 | Female | 0 | 0:LIVING | 61-70 | PD-1/PDL-1 |
| P-0004693 | P-0004693-T01-IM5 | 872 | 20 | LUAD | Matched | 74 | 9.787197 | Female | 2 | 1:DECEASED | >71 | PD-1/PDL-1 |
| P-0004701 | P-0004701-T01-IM5 | 595 | 20 | NSCLCPD | Matched | 53 | 19.57439 | Female | 30 | 0:LIVING | 50-60 | PD-1/PDL-1 |
| P-0004742 | P-0004742-T01-IM5 | 744 | 40 | LUSC | Matched | 69 | 8.808477 | Male | 18 | 0:LIVING | 61-70 | PD-1/PDL-1 |
| P-0004752 | P-0004752-T01-IM5 | 580 | 30 | LUAD | Matched | 86 | 0.97872 | Male | 29 | 0:LIVING | >71 | PD-1/PDL-1 |
| P-0004759 | P-0004759-T01-IM5 | 677 | 30 | LUAD | Matched | 63 | 1.957439 | Female | 2 | 1:DECEASED | 61-70 | PD-1/PDL-1 |
| P-0004827 | P-0004827-T01-IM5 | 662 | 20 | LUSC | Matched | 59 | 8.808477 | Male | 19 | 1:DECEASED | 50-60 | PD-1/PDL-1 |
| P-0004890 | P-0004890-T01-IM5 | 515 | 50 | LUAD | Matched | 69 | 10.76592 | Female | 18 | 0:LIVING | 61-70 | PD-1/PDL-1 |
| P-0004961 | P-0004961-T01-IM5 | 855 | 10 | LUAD | Matched | 68 | 0.97872 | Female | 9 | 1:DECEASED | 61-70 | PD-1/PDL-1 |
| P-0004996 | P-0004996-T02-IM5 | 1223 | 60 | LUSC | Matched | 57 | 2.936159 | Male | 46 | 1:DECEASED | 50-60 | Combo |
| P-0005048 | P-0005048-T01-IM5 | 965 | 20 | LUAD | Matched | 70 | 3.914879 | Female | 51 | 0:LIVING | 61-70 | Combo |
| P-0005115 | P-0005115-T01-IM5 | 539 | 70 | LUSC | Matched | 54 | 1.957439 | Male | 15 | 0:LIVING | 50-60 | PD-1/PDL-1 |
| P-0005252 | P-0005252-T01-IM5 | 56 | 30 | LUAD | Matched | 79 | 12.72336 | Female | 2 | 1:DECEASED | >71 | PD-1/PDL-1 |
| P-0005289 | P-0005289-T01-IM5 | 202 | 90 | LUAD | Matched | 68 | 9.787197 | Male | 5 | 1:DECEASED | 61-70 | PD-1/PDL-1 |
| P-0005295 | P-0005295-T01-IM5 | 839 | 50 | LUAD | Matched | 73 | 3.914879 | Male | 29 | 0:LIVING | >71 | PD-1/PDL-1 |
| P-0005320 | P-0005320-T01-IM5 | 709 | 10 | LUAD | Matched | 47 | 7.829758 | Male | 17 | 1:DECEASED | 31-50 | PD-1/PDL-1 |
| P-0005456 | P-0005456-T01-IM5 | 713 | 30 | LUSC | Matched | 77 | 5.872318 | Male | 2 | 1:DECEASED | >71 | PD-1/PDL-1 |
| P-0005464 | P-0005464-T01-IM5 | 896 | 30 | LUAD | Matched | 67 | 6.851038 | Female | 11 | 1:DECEASED | 61-70 | PD-1/PDL-1 |
| P-0005510 | P-0005510-T01-IM5 | 1384 | 30 | LUAD | Matched | 69 | 5.872318 | Female | 55 | 0:LIVING | 61-70 | PD-1/PDL-1 |
| P-0005553 | P-0005553-T01-IM5 | 1033 | 60 | LUAD | Matched | 49 | 4.893598 | Male | 1 | 1:DECEASED | 31-50 | PD-1/PDL-1 |
| P-0005579 | P-0005579-T01-IM5 | 936 | 30 | NSCLCPD | Matched | 56 | 1.957439 | Female | 1 | 1:DECEASED | 50-60 | PD-1/PDL-1 |
| P-0005629 | P-0005629-T02-IM5 | 472 | 10 | LUAD | Matched | 81 | 5.872318 | Female | 5 | 1:DECEASED | >71 | PD-1/PDL-1 |
| P-0005711 | P-0005711-T01-IM5 | 723 | 60 | LUAD | Matched | 47 | 2.936159 | Male | 6 | 1:DECEASED | 31-50 | PD-1/PDL-1 |
| P-0005721 | P-0005721-T01-IM5 | 622 | 20 | LUAD | Matched | 79 | 6.851038 | Male | 14 | 1:DECEASED | >71 | PD-1/PDL-1 |
| P-0005752 | P-0005752-T01-IM5 | 846 | 50 | LUAD | Matched | 66 | 3.914879 | Male | 14 | 1:DECEASED | 61-70 | PD-1/PDL-1 |
| P-0005792 | P-0005792-T01-IM5 | 1406 | 10 | LUSC | Matched | 59 | 10.76592 | Male | 10 | 1:DECEASED | 50-60 | PD-1/PDL-1 |
| P-0005848 | P-0005848-T01-IM5 | 248 | 60 | LUAD | Matched | 80 | 16.63823 | Female | 14 | 0:LIVING | >71 | PD-1/PDL-1 |
| P-0005851 | P-0005851-T01-IM5 | 608 | 30 | NSCLCPD | Matched | 80 | 20.55311 | Male | 18 | 0:LIVING | >71 | PD-1/PDL-1 |
| P-0005897 | P-0005897-T02-IM5 | 1061 | 20 | LUAD | Matched | 44 | 5.872318 | Male | 5 | 1:DECEASED | 31-50 | PD-1/PDL-1 |
| P-0005898 | P-0005898-T01-IM5 | 921 | 30 | LUSC | Matched | 71 | 3.914879 | Female | 21 | 0:LIVING | >71 | PD-1/PDL-1 |
| P-0005981 | P-0005981-T01-IM5 | 1121 | 70 | LUAD | Matched | 61 | 9.787197 | Male | 47 | 0:LIVING | 50-60 | PD-1/PDL-1 |
| P-0006046 | P-0006046-T01-IM5 | 716 | 70 | LUAD | Matched | 59 | 19.57439 | Male | 14 | 0:LIVING | 50-60 | PD-1/PDL-1 |
| P-0006047 | P-0006047-T02-IM5 | 1146 | 60 | LUAD | Matched | 63 | 1.957439 | Female | 24 | 0:LIVING | 61-70 | PD-1/PDL-1 |
| P-0006071 | P-0006071-T01-IM5 | 752 | 70 | LUAD | Matched | 52 | 19.57439 | Female | 19 | 0:LIVING | 50-60 | PD-1/PDL-1 |
| P-0006084 | P-0006084-T01-IM5 | 885 | 70 | LUAD | Matched | 62 | 6.851038 | Male | 21 | 1:DECEASED | 61-70 | PD-1/PDL-1 |
| P-0006114 | P-0006114-T01-IM5 | 1179 | 30 | LUAD | Matched | 54 | 2.936159 | Female | 7 | 1:DECEASED | 50-60 | PD-1/PDL-1 |
| P-0006221 | P-0006221-T01-IM5 | 753 | 10 | LUAD | Matched | 48 | 0 | Female | 13 | 1:DECEASED | 31-50 | PD-1/PDL-1 |
| P-0006268 | P-0006268-T01-IM5 | 591 | 20 | LUAD | Matched | 71 | 1.957439 | Male | 12 | 1:DECEASED | >71 | PD-1/PDL-1 |
| P-0006294 | P-0006294-T01-IM5 | 586 | 20 | LUAD | Matched | 63 | 3.914879 | Female | 15 | 1:DECEASED | 61-70 | PD-1/PDL-1 |
| P-0006321 | P-0006321-T01-IM5 | 351 | 70 | LUAD | Matched | 64 | 2.936159 | Male | 0 | 1:DECEASED | 61-70 | PD-1/PDL-1 |
| P-0006343 | P-0006343-T01-IM5 | 747 |  | LUAD | Matched | 61 | 5.872318 | Female | 28 | 0:LIVING | 50-60 | PD-1/PDL-1 |
| P-0006415 | P-0006415-T01-IM5 | 301 | 30 | LUAD | Matched | 85 | 4.893598 | Male | 12 | 1:DECEASED | >71 | PD-1/PDL-1 |
| P-0006516 | P-0006516-T01-IM5 | 888 | 10 | LUAD | Matched | 73 | 1.957439 | Male | 1 | 1:DECEASED | >71 | PD-1/PDL-1 |
| P-0006572 | P-0006572-T01-IM5 | 501 | 10 | LUAD | Matched | 73 | 0 | Male | 20 | 0:LIVING | >71 | PD-1/PDL-1 |
| P-0006582 | P-0006582-T01-IM5 | 814 | 30 | LUAD | Matched | 72 | 4.893598 | Female | 19 | 0:LIVING | >71 | PD-1/PDL-1 |
| P-0006642 | P-0006642-T01-IM5 | 1100 | 40 | LUAD | Matched | 67 | 20.55311 | Female | 14 | 1:DECEASED | 61-70 | PD-1/PDL-1 |
| P-0006674 | P-0006674-T01-IM5 | 723 | 50 | LUNE | Matched | 66 | 16.63823 | Female | 7 | 1:DECEASED | 61-70 | PD-1/PDL-1 |
| P-0006713 | P-0006713-T01-IM5 | 946 | 50 | LUAD | Matched | 52 | 45.02111 | Female | 23 | 0:LIVING | 50-60 | PD-1/PDL-1 |
| P-0006724 | P-0006724-T01-IM5 | 704 | 60 | NSCLCPD | Matched | 48 | 23.48927 | Female | 18 | 0:LIVING | 31-50 | PD-1/PDL-1 |
| P-0006728 | P-0006728-T01-IM5 | 647 | 20 | LUAD | Unmatched | 76 | 13.70208 | Female | 3 | 1:DECEASED | >71 | PD-1/PDL-1 |
| P-0006768 | P-0006768-T01-IM5 | 1424 | 50 | LUAD | Matched | 66 | 7.829758 | Male | 16 | 0:LIVING | 61-70 | PD-1/PDL-1 |
| P-0006895 | P-0006895-T01-IM5 | 882 |  | LUAD | Matched | 65 | 36.21263 | Male | 19 | 0:LIVING | 61-70 | PD-1/PDL-1 |
| P-0006954 | P-0006954-T01-IM5 | 691 |  | LUAD | Matched | 67 | 7.829758 | Female | 3 | 1:DECEASED | 61-70 | PD-1/PDL-1 |
| P-0006993 | P-0006993-T01-IM5 | 597 | 30 | LUAD | Matched | 68 | 7.829758 | Male | 3 | 1:DECEASED | 61-70 | PD-1/PDL-1 |
| P-0007051 | P-0007051-T01-IM5 | 715 | 30 | LUAD | Matched | 71 | 13.70208 | Male | 2 | 1:DECEASED | >71 | PD-1/PDL-1 |
| P-0007068 | P-0007068-T01-IM5 | 931 | 30 | LUSC | Matched | 65 | 8.808477 | Male | 6 | 1:DECEASED | 61-70 | PD-1/PDL-1 |
| P-0007083 | P-0007083-T02-IM6 | 520 | 50 | LUAD | Matched | 76 | 12.97047 | Male | 12 | 1:DECEASED | >71 | PD-1/PDL-1 |
| P-0007088 | P-0007088-T02-IM5 | 919 | 40 | LUAD | Matched | 45 | 4.893598 | Female | 32 | 1:DECEASED | 31-50 | PD-1/PDL-1 |
| P-0007099 | P-0007099-T01-IM5 | 963 | 30 | LUAD | Matched | 78 | 10.76592 | Female | 1 | 1:DECEASED | >71 | PD-1/PDL-1 |
| P-0007101 | P-0007101-T02-IM5 | 513 | 15 | LUAD | Matched | 76 | 11.74464 | Male | 6 | 1:DECEASED | >71 | PD-1/PDL-1 |
| P-0007114 | P-0007114-T01-IM5 | 970 | 10 | LUAD | Matched | 77 | 8.808477 | Male | 1 | 1:DECEASED | >71 | PD-1/PDL-1 |
| P-0007119 | P-0007119-T01-IM5 | 562 | 20 | LUAD | Matched | 61 | 22.51055 | Male | 21 | 0:LIVING | 61-70 | PD-1/PDL-1 |
| P-0007121 | P-0007121-T01-IM5 | 851 | 15 | LUAD | Matched | 59 | 7.829758 | Female | 17 | 0:LIVING | 50-60 | PD-1/PDL-1 |
| P-0007123 | P-0007123-T01-IM5 | 861 | 20 | LUAD | Matched | 67 | 8.808477 | Female | 24 | 0:LIVING | 61-70 | PD-1/PDL-1 |
| P-0007278 | P-0007278-T01-IM5 | 760 | 15 | LUAD | Matched | 57 | 7.829758 | Female | 8 | 1:DECEASED | 50-60 | PD-1/PDL-1 |
| P-0007418 | P-0007418-T01-IM5 | 519 | 70 | LUAD | Matched | 57 | 16.63823 | Female | 22 | 0:LIVING | 50-60 | PD-1/PDL-1 |
| P-0007420 | P-0007420-T01-IM5 | 673 |  | LUAD | Matched | 85 | 3.914879 | Female | 8 | 1:DECEASED | >71 | PD-1/PDL-1 |
| P-0007493 | P-0007493-T02-IM5 | 902 | 40 | LUAD | Matched | 75 | 18.59567 | Male | 13 | 0:LIVING | >71 | PD-1/PDL-1 |
| P-0007514 | P-0007514-T01-IM5 | 606 | 30 | LUSC | Matched | 73 | 3.914879 | Female | 2 | 1:DECEASED | >71 | PD-1/PDL-1 |
| P-0007525 | P-0007525-T01-IM5 | 413 | 15 | LUAD | Matched | 65 | 4.893598 | Female | 2 | 1:DECEASED | 61-70 | PD-1/PDL-1 |
| P-0007580 | P-0007580-T01-IM5 | 191 | 50 | LUAD | Matched | 63 | 2.936159 | Female | 10 | 1:DECEASED | 61-70 | PD-1/PDL-1 |
| P-0007582 | P-0007582-T01-IM5 | 780 | 50 | LUAD | Matched | 83 | 4.893598 | Male | 3 | 1:DECEASED | >71 | PD-1/PDL-1 |
| P-0007583 | P-0007583-T01-IM5 | 744 | 70 | LUAD | Matched | 70 | 13.70208 | Female | 7 | 1:DECEASED | 61-70 | PD-1/PDL-1 |
| P-0007597 | P-0007597-T01-IM5 | 1213 | 80 | LUAD | Matched | 73 | 15.65952 | Male | 5 | 1:DECEASED | >71 | PD-1/PDL-1 |
| P-0007619 | P-0007619-T01-IM5 | 785 | 40 | LUAD | Matched | 78 | 9.787197 | Male | 1 | 1:DECEASED | >71 | PD-1/PDL-1 |
| P-0007633 | P-0007633-T01-IM5 | 719 | 40 | LUAD | Matched | 79 | 2.936159 | Male | 48 | 0:LIVING | >71 | Combo |
| P-0007861 | P-0007861-T01-IM5 | 712 | 75 | LUAD | Matched | 77 | 2.936159 | Female | 12 | 1:DECEASED | >71 | PD-1/PDL-1 |
| P-0007879 | P-0007879-T01-IM5 | 827 | default | LUAD | Matched | 70 | 9.787197 | Female | 31 | 0:LIVING | 61-70 | PD-1/PDL-1 |
| P-0007916 | P-0007916-T01-IM5 | 699 |  | LUAD | Matched | 69 | 14.6808 | Male | 22 | 0:LIVING | 61-70 | PD-1/PDL-1 |
| P-0007977 | P-0007977-T01-IM5 | 420 | 10 | NSCLC | Matched | 70 | 5.872318 | Female | 3 | 1:DECEASED | 61-70 | PD-1/PDL-1 |
| P-0007978 | P-0007978-T01-IM5 | 707 | 10 | LUAD | Matched | 50 | 10.76592 | Female | 6 | 1:DECEASED | 31-50 | PD-1/PDL-1 |
| P-0008161 | P-0008161-T01-IM5 | 1182 | 20 | LUAD | Matched | 82 | 4.893598 | Male | 3 | 0:LIVING | >71 | PD-1/PDL-1 |
| P-0008176 | P-0008176-T01-IM5 | 698 |  | LUAD | Matched | 72 | 3.914879 | Female | 23 | 0:LIVING | >71 | PD-1/PDL-1 |
| P-0008239 | P-0008239-T01-IM5 | 465 | 30 | LUSC | Matched | 74 | 15.65952 | Female | 15 | 1:DECEASED | >71 | PD-1/PDL-1 |
| P-0008295 | P-0008295-T01-IM5 | 1118 | 30 | LUAD | Matched | 58 | 5.872318 | Male | 14 | 0:LIVING | 50-60 | PD-1/PDL-1 |
| P-0008322 | P-0008322-T01-IM5 | 788 | 30 | LUAD | Matched | 67 | 2.936159 | Male | 29 | 0:LIVING | 61-70 | PD-1/PDL-1 |
| P-0008332 | P-0008332-T01-IM5 | 509 | 90 | LUAD | Matched | 57 | 3.914879 | Female | 26 | 0:LIVING | 50-60 | PD-1/PDL-1 |
| P-0008438 | P-0008438-T01-IM5 | 737 | 70 | LUAD | Matched | 63 | 2.936159 | Male | 0 | 0:LIVING | 61-70 | PD-1/PDL-1 |
| P-0008442 | P-0008442-T01-IM5 | 501 | 30 | LUAD | Matched | 65 | 4.893598 | Female | 11 | 1:DECEASED | 61-70 | PD-1/PDL-1 |
| P-0008678 | P-0008678-T01-IM5 | 1066 | 60 | LUAD | Matched | 73 | 7.829758 | Female | 25 | 0:LIVING | >71 | PD-1/PDL-1 |
| P-0008765 | P-0008765-T01-IM5 | 1040 | 20 | LUAD | Matched | 60 | 7.829758 | Male | 3 | 0:LIVING | 50-60 | PD-1/PDL-1 |
| P-0008786 | P-0008786-T01-IM5 | 1174 | 30 | LUAD | Matched | 65 | 4.893598 | Male | 24 | 0:LIVING | 61-70 | PD-1/PDL-1 |
| P-0008848 | P-0008848-T01-IM5 | 1029 | 60 | LUNE | Matched | 76 | 5.872318 | Male | 5 | 1:DECEASED | >71 | PD-1/PDL-1 |
| P-0008851 | P-0008851-T01-IM5 | 1102 | 90 | NSCLC | Matched | 63 | 7.829758 | Male | 3 | 1:DECEASED | 61-70 | PD-1/PDL-1 |
| P-0008874 | P-0008874-T01-IM5 | 923 | 30 | LUAD | Matched | 58 | 10.76592 | Male | 1 | 1:DECEASED | 50-60 | PD-1/PDL-1 |
| P-0008922 | P-0008922-T01-IM5 | 832 | 40 | LUAD | Matched | 67 | 2.936159 | Male | 1 | 1:DECEASED | 61-70 | PD-1/PDL-1 |
| P-0008935 | P-0008935-T01-IM5 | 543 | 80 | LUAD | Matched | 75 | 10.76592 | Female | 5 | 1:DECEASED | >71 | PD-1/PDL-1 |
| P-0008944 | P-0008944-T01-IM5 | 718 | 40 | LUAD | Matched | 47 | 10.76592 | Female | 11 | 1:DECEASED | 31-50 | PD-1/PDL-1 |
| P-0009017 | P-0009017-T01-IM5 | 1097 | 40 | LUAD | Matched | 77 | 33.27647 | Male | 10 | 0:LIVING | >71 | PD-1/PDL-1 |
| P-0009020 | P-0009020-T01-IM5 | 449 | 20 | LUAD | Matched | 74 | 12.72336 | Female | 8 | 1:DECEASED | >71 | PD-1/PDL-1 |
| P-0009068 | P-0009068-T01-IM5 | 1030 | 50 | NSCLCPD | Matched | 74 | 19.57439 | Female | 22 | 0:LIVING | >71 | PD-1/PDL-1 |
| P-0009080 | P-0009080-T01-IM5 | 973 | 80 | LUSC | Matched | 74 | 5.872318 | Male | 7 | 1:DECEASED | >71 | PD-1/PDL-1 |
| P-0009161 | P-0009161-T01-IM5 | 912 | 60 | LUAD | Matched | 63 | 10.76592 | Female | 13 | 0:LIVING | 61-70 | PD-1/PDL-1 |
| P-0009256 | P-0009256-T01-IM5 | 828 |  | LUAD | Matched | 76 | 9.787197 | Female | 26 | 0:LIVING | >71 | PD-1/PDL-1 |
| P-0009274 | P-0009274-T01-IM5 | 1002 |  | LUAD | Matched | 77 | 3.914879 | Female | 7 | 1:DECEASED | >71 | Combo |
| P-0009293 | P-0009293-T01-IM5 | 558 | 40 | LUAD | Matched | 81 | 21.53183 | Female | 25 | 0:LIVING | >71 | PD-1/PDL-1 |
| P-0009319 | P-0009319-T01-IM5 | 919 | 60 | LUAD | Matched | 64 | 1.957439 | Male | 35 | 0:LIVING | 61-70 | Combo |
| P-0009370 | P-0009370-T01-IM5 | 827 | 30 | LUAD | Matched | 67 | 8.808477 | Female | 1 | 0:LIVING | 61-70 | PD-1/PDL-1 |
| P-0009379 | P-0009379-T01-IM5 | 1224 | N/A | LUAD | Matched | 69 | 9.787197 | Male | 1 | 1:DECEASED | 61-70 | PD-1/PDL-1 |
| P-0009419 | P-0009419-T01-IM5 | 606 |  | LUAD | Matched | 61 | 12.72336 | Male | 20 | 0:LIVING | 50-60 | PD-1/PDL-1 |
| P-0009426 | P-0009426-T01-IM5 | 227 | 20 | LUAD | Matched | 65 | 9.787197 | Female | 11 | 1:DECEASED | 61-70 | PD-1/PDL-1 |
| P-0009431 | P-0009431-T01-IM5 | 638 | 70 | LUAD | Matched | 74 | 8.808477 | Female | 1 | 1:DECEASED | >71 | PD-1/PDL-1 |
| P-0009434 | P-0009434-T01-IM5 | 560 | 10 | LUAD | Matched | 58 | 4.893598 | Male | 0 | 0:LIVING | 50-60 | PD-1/PDL-1 |
| P-0009469 | P-0009469-T01-IM5 | 696 | 60 | LUAD | Matched | 32 | 2.936159 | Female | 3 | 0:LIVING | 31-50 | PD-1/PDL-1 |
| P-0009473 | P-0009473-T01-IM5 | 709 | 20 | LUAS | Matched | 80 | 8.808477 | Male | 23 | 0:LIVING | >71 | PD-1/PDL-1 |
| P-0009476 | P-0009476-T01-IM5 | 717 | 60 | LUAD | Matched | 58 | 7.829758 | Female | 5 | 1:DECEASED | 50-60 | PD-1/PDL-1 |
| P-0009483 | P-0009483-T01-IM5 | 802 |  | LUAD | Matched | 82 | 9.787197 | Female | 9 | 1:DECEASED | >71 | PD-1/PDL-1 |
| P-0009501 | P-0009501-T01-IM5 | 777 | 40 | LUSC | Matched | 73 | 5.872318 | Male | 23 | 1:DECEASED | >71 | PD-1/PDL-1 |
| P-0009505 | P-0009505-T01-IM5 | 1088 | 30 | LUAD | Matched | 63 | 2.936159 | Female | 3 | 1:DECEASED | 61-70 | PD-1/PDL-1 |
| P-0009526 | P-0009526-T01-IM5 | 697 | 60 | LUSC | Matched | 69 | 11.74464 | Male | 5 | 1:DECEASED | 61-70 | PD-1/PDL-1 |
| P-0009532 | P-0009532-T01-IM5 | 1032 | 80 | LUAD | Matched | 59 | 10.76592 | Male | 2 | 1:DECEASED | 50-60 | PD-1/PDL-1 |
| P-0009539 | P-0009539-T01-IM5 | 1065 | 60 | LUAD | Matched | 73 | 0 | Female | 1 | 1:DECEASED | >71 | PD-1/PDL-1 |
| P-0009586 | P-0009586-T01-IM5 | 1196 |  | LUAD | Matched | 56 | 11.74464 | Female | 24 | 0:LIVING | 50-60 | PD-1/PDL-1 |
| P-0009638 | P-0009638-T01-IM5 | 1002 | 20 | LUAD | Matched | 82 | 18.59567 | Male | 2 | 1:DECEASED | >71 | PD-1/PDL-1 |
| P-0009655 | P-0009655-T01-IM5 | 948 | 60 | LUAD | Matched | 71 | 2.936159 | Female | 56 | 0:LIVING | 61-70 | Combo |
| P-0009660 | P-0009660-T01-IM5 | 606 | 70 | LUAD | Matched | 61 | 23.48927 | Male | 20 | 0:LIVING | 61-70 | PD-1/PDL-1 |
| P-0009714 | P-0009714-T01-IM5 | 1544 | 70 | LUAD | Matched | 60 | 19.57439 | Male | 5 | 1:DECEASED | 50-60 | PD-1/PDL-1 |
| P-0009762 | P-0009762-T01-IM5 | 963 | 30 | LUAD | Matched | 71 | 7.829758 | Male | 16 | 1:DECEASED | >71 | PD-1/PDL-1 |
| P-0009764 | P-0009764-T01-IM5 | 893 | 40 | LUAD | Matched | 51 | 22.51055 | Female | 16 | 0:LIVING | 31-50 | PD-1/PDL-1 |
| P-0009833 | P-0009833-T01-IM5 | 972 | 40 | LUAD | Matched | 54 | 32.29775 | Female | 20 | 0:LIVING | 50-60 | PD-1/PDL-1 |
| P-0009915 | P-0009915-T01-IM5 | 882 | 60 | LUSC | Matched | 59 | 7.829758 | Male | 7 | 1:DECEASED | 50-60 | PD-1/PDL-1 |
| P-0009924 | P-0009924-T01-IM5 | 982 | 70 | LUAD | Matched | 79 | 17.61695 | Male | 13 | 0:LIVING | >71 | PD-1/PDL-1 |
| P-0009959 | P-0009959-T01-IM5 | 1367 | 20 | LUAD | Matched | 66 | 2.936159 | Male | 13 | 1:DECEASED | 61-70 | PD-1/PDL-1 |
| P-0010013 | P-0010013-T01-IM5 | 269 | 10 | NSCLC | Matched | 77 | 1.957439 | Male | 3 | 1:DECEASED | >71 | PD-1/PDL-1 |
| P-0010024 | P-0010024-T01-IM5 | 1238 | 20 | LUSC | Matched | 81 | 3.914879 | Male | 27 | 0:LIVING | >71 | PD-1/PDL-1 |
| P-0010025 | P-0010025-T01-IM5 | 1025 | 40 | LUAD | Matched | 58 | 10.76592 | Female | 10 | 1:DECEASED | 50-60 | PD-1/PDL-1 |
| P-0010065 | P-0010065-T01-IM5 | 589 | 10 | NSCLC | Matched | 40 | 1.957439 | Male | 9 | 0:LIVING | 31-50 | PD-1/PDL-1 |
| P-0010213 | P-0010213-T01-IM5 | 631 | 80 | LUAD | Matched | 51 | 15.65952 | Female | 25 | 0:LIVING | 31-50 | PD-1/PDL-1 |
| P-0010215 | P-0010215-T01-IM5 | 856 | 20 | LUAD | Matched | 74 | 6.851038 | Female | 3 | 1:DECEASED | >71 | PD-1/PDL-1 |
| P-0010256 | P-0010256-T01-IM5 | 556 | 40 | LUAD | Matched | 73 | 3.914879 | Female | 21 | 1:DECEASED | >71 | PD-1/PDL-1 |
| P-0010257 | P-0010257-T01-IM5 | 961 | 30 | LUAD | Matched | 55 | 9.787197 | Female | 0 | 1:DECEASED | 50-60 | PD-1/PDL-1 |
| P-0010355 | P-0010355-T01-IM5 | 825 | 30 | LUAD | Matched | 69 | 6.851038 | Female | 1 | 1:DECEASED | 61-70 | PD-1/PDL-1 |
| P-0010362 | P-0010362-T01-IM5 | 784 | 10 | LUAD | Matched | 60 | 2.936159 | Female | 3 | 1:DECEASED | 50-60 | PD-1/PDL-1 |
| P-0010385 | P-0010385-T01-IM5 | 1185 | 60 | LUAD | Matched | 72 | 7.829758 | Male | 0 | 1:DECEASED | >71 | PD-1/PDL-1 |
| P-0010389 | P-0010389-T01-IM5 | 1039 | 20 | LUSC | Matched | 69 | 0 | Male | 9 | 1:DECEASED | 61-70 | PD-1/PDL-1 |
| P-0010636 | P-0010636-T01-IM5 | 1073 | 70 | LUSC | Matched | 68 | 6.851038 | Male | 16 | 1:DECEASED | 61-70 | PD-1/PDL-1 |
| P-0010645 | P-0010645-T01-IM5 | 699 | 20 | LUSC | Matched | 53 | 3.914879 | Male | 3 | 1:DECEASED | 50-60 | PD-1/PDL-1 |
| P-0010648 | P-0010648-T01-IM5 | 553 | 40 | LUAD | Matched | 73 | 1.957439 | Male | 18 | 1:DECEASED | >71 | PD-1/PDL-1 |
| P-0010715 | P-0010715-T01-IM5 | 1357 | 50 | LUNE | Matched | 67 | 49.9147 | Male | 15 | 0:LIVING | 61-70 | Combo |
| P-0010736 | P-0010736-T01-IM5 | 733 | 40 | LUAD | Matched | 80 | 25.44671 | Male | 18 | 1:DECEASED | >71 | PD-1/PDL-1 |
| P-0010760 | P-0010760-T01-IM5 | 1001 | 20 | LUAD | Matched | 51 | 4.893598 | Male | 22 | 0:LIVING | 50-60 | PD-1/PDL-1 |
| P-0010840 | P-0010840-T02-IM5 | 313 | 20 | LUAD | Matched | 55 | 10.76592 | Female | 2 | 1:DECEASED | 50-60 | PD-1/PDL-1 |
| P-0010842 | P-0010842-T01-IM5 | 1031 | 20 | LUAD | Matched | NA | 0 | Male | 0 | 1:DECEASED | >71 | PD-1/PDL-1 |
| P-0010855 | P-0010855-T01-IM5 | 817 | 40 | LUAD | Matched | 74 | 6.851038 | Female | 7 | 1:DECEASED | >71 | PD-1/PDL-1 |
| P-0010891 | P-0010891-T01-IM5 | 881 | 15 | LUAD | Matched | 42 | 0.97872 | Female | 2 | 1:DECEASED | 31-50 | Combo |
| P-0010945 | P-0010945-T01-IM5 | 745 | 15 | LUAD | Matched | 52 | 0 | Male | 1 | 1:DECEASED | 50-60 | PD-1/PDL-1 |
| P-0010986 | P-0010986-T01-IM5 | 1215 | 40 | LUAD | Matched | 55 | 7.829758 | Female | 2 | 1:DECEASED | 50-60 | PD-1/PDL-1 |
| P-0011012 | P-0011012-T01-IM5 | 448 | 20 | LUAD | Matched | 74 | 3.914879 | Male | 3 | 1:DECEASED | >71 | PD-1/PDL-1 |
| P-0011030 | P-0011030-T01-IM5 | 430 | 30 | LUAD | Matched | 70 | 0.97872 | Male | 1 | 1:DECEASED | 61-70 | PD-1/PDL-1 |
| P-0011033 | P-0011033-T01-IM5 | 806 | 75 | LUAD | Matched | 62 | 1.957439 | Female | 11 | 1:DECEASED | 61-70 | PD-1/PDL-1 |
| P-0011036 | P-0011036-T01-IM5 | 1170 | 30 | LUAD | Matched | 56 | 11.74464 | Male | 4 | 1:DECEASED | 50-60 | PD-1/PDL-1 |
| P-0011180 | P-0011180-T01-IM5 | 913 | 20 | LUAD | Matched | 61 | 1.957439 | Female | 7 | 0:LIVING | 50-60 | PD-1/PDL-1 |
| P-0011192 | P-0011192-T01-IM5 | 920 | 40 | LUAD | Matched | 74 | 10.76592 | Female | 2 | 0:LIVING | >71 | PD-1/PDL-1 |
| P-0011244 | P-0011244-T01-IM5 | 1071 | 10 | LUSC | Matched | 77 | 2.936159 | Male | 3 | 1:DECEASED | >71 | PD-1/PDL-1 |
| P-0011378 | P-0011378-T01-IM5 | 904 | 30 | NSCLCPD | Matched | 68 | 8.808477 | Male | 2 | 1:DECEASED | 61-70 | PD-1/PDL-1 |
| P-0011453 | P-0011453-T01-IM5 | 818 | 20 | LUAD | Matched | 66 | 5.872318 | Male | 1 | 1:DECEASED | 61-70 | PD-1/PDL-1 |
| P-0011499 | P-0011499-T01-IM5 | 884 | 30 | LUAD | Matched | 71 | 7.829758 | Female | 6 | 1:DECEASED | 61-70 | PD-1/PDL-1 |
| P-0011508 | P-0011508-T01-IM5 | 954 | 60 | LUNE | Matched | 62 | 6.851038 | Male | 1 | 1:DECEASED | 61-70 | PD-1/PDL-1 |
| P-0011535 | P-0011535-T01-IM5 | 993 | 50 | LUAD | Matched | 73 | 2.936159 | Female | 8 | 1:DECEASED | >71 | PD-1/PDL-1 |
| P-0011541 | P-0011541-T01-IM5 | 353 | 20 | LUAD | Matched | 77 | 3.914879 | Female | 13 | 0:LIVING | >71 | PD-1/PDL-1 |
| P-0011546 | P-0011546-T01-IM5 | 872 | 60 | LUNE | Matched | 62 | 9.787197 | Female | 3 | 0:LIVING | 61-70 | PD-1/PDL-1 |
| P-0011559 | P-0011559-T01-IM5 | 883 | 10 | LUAD | Matched | 73 | 3.914879 | Male | 7 | 1:DECEASED | >71 | PD-1/PDL-1 |
| P-0012049 | P-0012049-T01-IM5 | 889 | 60 | LUAD | Matched | 68 | 10.76592 | Female | 11 | 0:LIVING | 61-70 | PD-1/PDL-1 |
| P-0012341 | P-0012341-T01-IM5 | 885 | 30 | LUAD | Matched | 72 | 5.872318 | Female | 3 | 1:DECEASED | >71 | PD-1/PDL-1 |
| P-0012427 | P-0012427-T01-IM5 | 456 | 20 | LUAD | Matched | 67 | 5.872318 | Male | 7 | 1:DECEASED | 61-70 | PD-1/PDL-1 |
| P-0012629 | P-0012629-T01-IM5 | 734 | 50 | NSCLCPD | Matched | 82 | 2.936159 | Male | 18 | 1:DECEASED | >71 | PD-1/PDL-1 |
| P-0012678 | P-0012678-T01-IM5 | 1144 | 40 | LUSC | Matched | 73 | 23.48927 | Male | 20 | 0:LIVING | >71 | PD-1/PDL-1 |
| P-0012729 | P-0012729-T01-IM5 | 906 | 40 | LUAD | Matched | 69 | 6.851038 | Male | 9 | 1:DECEASED | 61-70 | PD-1/PDL-1 |
| P-0012817 | P-0012817-T01-IM5 | 977 | 20 | LUAD | Matched | 55 | 33.27647 | Male | 18 | 0:LIVING | 50-60 | PD-1/PDL-1 |
| P-0012846 | P-0012846-T01-IM5 | 682 | 10 | NSCLCPD | Matched | 59 | 7.829758 | Female | 20 | 0:LIVING | 50-60 | PD-1/PDL-1 |
| P-0012849 | P-0012849-T01-IM5 | 918 | 70 | LUAD | Matched | 67 | 9.787197 | Male | 2 | 1:DECEASED | 61-70 | PD-1/PDL-1 |
| P-0012866 | P-0012866-T01-IM5 | 802 | 30 | LUAD | Matched | 51 | 5.872318 | Female | 19 | 1:DECEASED | 31-50 | PD-1/PDL-1 |
| P-0012908 | P-0012908-T01-IM5 | 1007 | 30 | LUAD | Matched | 80 | 6.851038 | Male | 13 | 1:DECEASED | >71 | PD-1/PDL-1 |
| P-0012925 | P-0012925-T01-IM5 | 248 | 80 | LUSC | Matched | 74 | 5.872318 | Male | 10 | 1:DECEASED | >71 | PD-1/PDL-1 |
| P-0012931 | P-0012931-T01-IM5 | 765 | 30 | LUSC | Matched | 72 | 9.787197 | Male | 2 | 0:LIVING | >71 | PD-1/PDL-1 |
| P-0012993 | P-0012993-T01-IM5 | 987 | 60 | LUAD | Matched | 87 | 5.872318 | Male | 4 | 1:DECEASED | >71 | PD-1/PDL-1 |
| P-0013063 | P-0013063-T01-IM5 | 1085 | 30 | LUSC | Matched | 57 | 10.76592 | Male | 0 | 1:DECEASED | 50-60 | PD-1/PDL-1 |
| P-0013189 | P-0013189-T01-IM5 | 1107 | 80 | LUAD | Matched | 58 | 1.957439 | Female | 20 | 0:LIVING | 50-60 | PD-1/PDL-1 |
| P-0013233 | P-0013233-T01-IM5 | 869 | 10 | LUAD | Matched | 70 | 7.829758 | Female | 13 | 0:LIVING | 61-70 | PD-1/PDL-1 |
| P-0013656 | P-0013656-T01-IM5 | 1049 | 20 | LUAD | Matched | 55 | 3.914879 | Female | 5 | 1:DECEASED | 50-60 | PD-1/PDL-1 |
| P-0014172 | P-0014172-T01-IM5 | 365 | 20 | LUAD | Matched | 83 | 17.61695 | Male | 9 | 1:DECEASED | >71 | PD-1/PDL-1 |
| P-0014207 | P-0014207-T01-IM6 | 619 | 10 | LUAD | Matched | 79 | 6.917585 | Male | 4 | 1:DECEASED | >71 | PD-1/PDL-1 |
| P-0014269 | P-0014269-T01-IM6 | 655 | 20 | NSCLCPD | Matched | 75 | 0.864698 | Male | 9 | 1:DECEASED | >71 | PD-1/PDL-1 |
| P-0014328 | P-0014328-T01-IM6 | 1288 | 80 | LUNE | Matched | 61 | 4.32349 | Female | 1 | 1:DECEASED | 61-70 | Combo |
| P-0014451 | P-0014451-T01-IM6 | 375 | 20 | LUAD | Matched | 65 | 9.511679 | Female | 8 | 1:DECEASED | 61-70 | PD-1/PDL-1 |
| P-0014513 | P-0014513-T01-IM6 | 1094 | 30 | NSCLCPD | Matched | 69 | 2.594094 | Female | 3 | 0:LIVING | 61-70 | PD-1/PDL-1 |
| P-0014529 | P-0014529-T01-IM6 | 1150 | 30 | LUSC | Matched | 65 | 21.61745 | Female | 16 | 0:LIVING | 61-70 | PD-1/PDL-1 |
| P-0014562 | P-0014562-T01-IM6 | 1091 | 30 | NSCLCPD | Matched | 71 | 12.10577 | Male | 6 | 0:LIVING | 61-70 | PD-1/PDL-1 |
| P-0014599 | P-0014599-T01-IM6 | 540 | 60 | NSCLC | Matched | 63 | 14.69987 | Male | 17 | 0:LIVING | 61-70 | PD-1/PDL-1 |
| P-0014736 | P-0014736-T01-IM6 | 990 | 10 | LUAD | Matched | 63 | 5.188189 | Male | 1 | 1:DECEASED | 61-70 | PD-1/PDL-1 |
| P-0014772 | P-0014772-T01-IM6 | 529 | 30 | LUAD | Matched | 74 | 8.646981 | Female | 5 | 1:DECEASED | >71 | PD-1/PDL-1 |
| P-0014786 | P-0014786-T01-IM6 | 446 | 50 | NSCLCPD | Matched | 65 | 11.24108 | Female | 6 | 1:DECEASED | 61-70 | PD-1/PDL-1 |
| P-0014818 | P-0014818-T01-IM6 | 1048 | 20 | LUAD | Matched | 83 | 28.53504 | Male | 12 | 1:DECEASED | >71 | PD-1/PDL-1 |
| P-0015047 | P-0015047-T01-IM6 | 661 | 40 | LUAD | Matched | 76 | 12.97047 | Female | 15 | 0:LIVING | >71 | PD-1/PDL-1 |
| P-0015078 | P-0015078-T01-IM6 | 809 | 50 | LUAD | Matched | 71 | 7.782283 | Female | 25 | 0:LIVING | 61-70 | PD-1/PDL-1 |
| P-0015085 | P-0015085-T01-IM6 | 778 | 20 | LUAD | Matched | 49 | 6.917585 | Female | 10 | 1:DECEASED | 31-50 | PD-1/PDL-1 |
| P-0015177 | P-0015177-T01-IM6 | 857 | 30 | LUPC | Matched | 73 | 7.782283 | Male | 1 | 1:DECEASED | >71 | PD-1/PDL-1 |
| P-0015214 | P-0015214-T01-IM6 | 615 | 30 | LUAD | Matched | 74 | 24.21155 | Female | 13 | 1:DECEASED | >71 | PD-1/PDL-1 |
| P-0015222 | P-0015222-T01-IM6 | 799 | 30 | LUAD | Matched | 62 | 7.782283 | Male | 0 | 1:DECEASED | 61-70 | PD-1/PDL-1 |
| P-0015259 | P-0015259-T01-IM6 | 183 | 30 | LUAD | Matched | 88 | 2.594094 | Male | 14 | 0:LIVING | >71 | PD-1/PDL-1 |
| P-0015296 | P-0015296-T01-IM6 | 893 | 70 | NSCLCPD | Matched | 69 | 19.88806 | Male | 15 | 0:LIVING | 61-70 | PD-1/PDL-1 |
| P-0015348 | P-0015348-T01-IM6 | 788 | 20 | LUAD | Matched | 63 | 10.37638 | Male | 15 | 0:LIVING | 61-70 | PD-1/PDL-1 |
| P-0015531 | P-0015531-T01-IM6 | 786 | 60 | LUSC | Matched | 54 | 4.32349 | Female | 9 | 1:DECEASED | 50-60 | PD-1/PDL-1 |
| P-0015556 | P-0015556-T01-IM6 | 749 | 30 | LUAD | Matched | 67 | 4.32349 | Male | 30 | 0:LIVING | 61-70 | PD-1/PDL-1 |
| P-0015593 | P-0015593-T01-IM6 | 836 | 30 | LUAD | Matched | 69 | 4.32349 | Female | 14 | 0:LIVING | 61-70 | PD-1/PDL-1 |
| P-0015600 | P-0015600-T01-IM6 | 919 | 70 | LUAD | Matched | 50 | 4.32349 | Female | 13 | 1:DECEASED | 31-50 | PD-1/PDL-1 |
| P-0015648 | P-0015648-T01-IM6 | 160 | 10 | LUSC | Matched | 67 | 4.32349 | Male | 20 | 0:LIVING | 61-70 | PD-1/PDL-1 |
| P-0015758 | P-0015758-T01-IM6 | 942 | 40 | NSCLC | Matched | 89 | 5.188189 | Female | 13 | 0:LIVING | >71 | PD-1/PDL-1 |
| P-0015780 | P-0015780-T01-IM6 | 994 | 40 | LUAD | Matched | 53 | 6.052887 | Male | 18 | 0:LIVING | 50-60 | PD-1/PDL-1 |
| P-0015785 | P-0015785-T01-IM6 | 1215 | 40 | LUAD | Matched | 57 | 22.48215 | Female | 9 | 1:DECEASED | 50-60 | PD-1/PDL-1 |
| P-0015937 | P-0015937-T01-IM6 | 311 | 30 | LUAD | Matched | 74 | 10.37638 | Female | 15 | 0:LIVING | >71 | PD-1/PDL-1 |
| P-0015955 | P-0015955-T01-IM6 | 509 | 20 | LUAD | Matched | 75 | 6.917585 | Female | 14 | 1:DECEASED | >71 | PD-1/PDL-1 |
| P-0015984 | P-0015984-T01-IM6 | 737 | 50 | LUSC | Matched | 67 | 24.21155 | Female | 6 | 1:DECEASED | 61-70 | PD-1/PDL-1 |
| P-0015987 | P-0015987-T01-IM6 | 929 | 60 | LUAD | Matched | 71 | 7.782283 | Female | 10 | 1:DECEASED | 61-70 | PD-1/PDL-1 |
| P-0016033 | P-0016033-T01-IM6 | 813 | 40 | LUAD | Matched | 73 | 28.53504 | Male | 15 | 0:LIVING | >71 | PD-1/PDL-1 |
| P-0016046 | P-0016046-T01-IM6 | 874 | 30 | LUAD | Matched | 69 | 5.188189 | Female | 16 | 0:LIVING | 61-70 | PD-1/PDL-1 |
| P-0016174 | P-0016174-T01-IM6 | 811 | 20 | LUAD | Matched | 73 | 12.97047 | Female | 14 | 0:LIVING | >71 | PD-1/PDL-1 |
| P-0016175 | P-0016175-T01-IM6 | 806 | 30 | LUAD | Matched | 77 | 14.69987 | Female | 1 | 1:DECEASED | >71 | PD-1/PDL-1 |
| P-0016185 | P-0016185-T01-IM6 | 594 | 10 | LUAD | Matched | 73 | 6.052887 | Male | 9 | 1:DECEASED | >71 | PD-1/PDL-1 |
| P-0016284 | P-0016284-T01-IM6 | 686 | 20 | LUAD | Matched | 80 | 0.864698 | Male | 20 | 0:LIVING | >71 | PD-1/PDL-1 |
| P-0016290 | P-0016290-T01-IM6 | 775 | 40 | LUAD | Matched | 83 | 8.646981 | Female | 2 | 1:DECEASED | >71 | PD-1/PDL-1 |
| P-0016308 | P-0016308-T01-IM6 | 1220 | 20 | NSCLC | Matched | 71 | 14.69987 | Female | 14 | 1:DECEASED | 61-70 | PD-1/PDL-1 |
| P-0016481 | P-0016481-T01-IM6 | 311 | 20 | NSCLC | Matched | 62 | 19.88806 | Male | 6 | 1:DECEASED | 61-70 | PD-1/PDL-1 |
| P-0016542 | P-0016542-T01-IM6 | 878 | 40 | LUAS | Matched | 80 | 19.02336 | Male | 1 | 1:DECEASED | >71 | PD-1/PDL-1 |
| P-0016614 | P-0016614-T01-IM6 | 757 | 20 | LUAD | Matched | 60 | 7.782283 | Female | 4 | 1:DECEASED | 50-60 | PD-1/PDL-1 |
| P-0016646 | P-0016646-T01-IM6 | 1126 | 20 | LUAD | Matched | 77 | 9.511679 | Male | 4 | 1:DECEASED | >71 | PD-1/PDL-1 |
| P-0016782 | P-0016782-T01-IM6 | 979 | 10 | LUAD | Matched | 61 | 21.61745 | Female | 3 | 0:LIVING | 50-60 | PD-1/PDL-1 |
| P-0016875 | P-0016875-T01-IM6 | 770 | 10 | LUAD | Matched | 89 | 5.188189 | Female | 1 | 0:LIVING | >71 | PD-1/PDL-1 |
| P-0016910 | P-0016910-T01-IM6 | 1040 | 10 | LUAD | Matched | 70 | 2.594094 | Female | 13 | 0:LIVING | 61-70 | PD-1/PDL-1 |
